# 7-Geranyloxycinnamic Acid Isolated from *Melicope lunu-ankenda* Leaves Perturbs Colon Cancer and Breast Cancer Cell Lines’ Growth via Induction of Apoptotic Pathway

**DOI:** 10.3390/molecules28083612

**Published:** 2023-04-21

**Authors:** Enas Mohamed Eliaser, Najihah Mohd. Hashim, Yaya Rukayadi, Ahmad Faizal Abdull Razis

**Affiliations:** 1 UPM-MAKNA Cancer Research Laboratory, Institute of Bioscience, Universiti Putra Malaysia, Serdang 43400, Malaysia; 2 Department of Biology, Faculty of Science, El-Mergib University, El Khums, Libya; 3 Department of Pharmaceutical Chemistry, Faculty of Pharmacy, Universiti Malaya, Kuala Lumpur 50603, Malaysia; 4 Center of Natural Product Research and Drug Discovery (CENAR), Universiti Malaya, Kuala Lumpur 50603, Malaysia; 5 Department of Food Science, Faculty of Food Science and Technology, Universiti Putra Malaysia, Serdang 43400, Malaysia; 6 Natural Medicines and Products Research Laboratory, Institute of Bioscience, Universiti Putra Malaysia, Serdang 43400, Malaysia

**Keywords:** cancer, *Melicope lunu-ankenda*, 7-geranyloxycinnamic acid, cytotoxicity

## Abstract

Globally, breast cancer is the most prevalent form of cancer in women and there is a need for alternative therapies such as plant-derived compounds with low systemic toxicity and selective toxicity to cancer cells. The aim of this study is to assess the cytotoxicity effects of 7-geranyloxycinnamic acid isolated from leaves of *Melicope lunu-ankenda*, a traditional medicinal plant, on the human breast cancer cell lines. Dried leaf powder was used for the preparation of different crude extracts using different solvents of increasing order of polarity. The structure of the isolated compound from the petroleum ether extract was elucidated by ^1^H and ^13^C NMR, LC-MS, and DIP−MS spectroscopy. The cytotoxic activity of the crude extract and 7-geranyloxycinnamic acid analyzed using MTT assay. Apoptotic analysis was evaluated using Annexin V-PI staining, AO/PI staining, intracellular ROS measurement, and measurement of activities of caspases 3/7, 8, and 9. Crude extracts and the isolated pure compound showed significant cytotoxicity against tested cancer cell lines. 7-geranyloxycinnamic acid was found to exert significant cytotoxic effects against breast cancer cell lines such as the MCF-7 and MDA-MB-231 cell lines. The cytotoxic effects are attributed to its ability to induce apoptosis via accumulation of ROS and activation of caspases in both breast cancer cell lines. The pure compound, 7-geranyloxycinnamic acid isolated from the leaves of *M. lunu-ankenda*, can exert significant cytotoxic effects against breast cancer cell lines without affecting the normal cells.

## 1. Introduction

Breast cancer is a serious public health concern in both developed and developing nations and it is the most frequent cause of cancer-related deaths in women [1]. The incidence and the mortality rates of breast cancer have increased in the last few decades. According to the Global Estimates of Cancer (GLOBOCAN) 2020, breast cancer is the first most frequently diagnosed cancer, followed by lung and colon cancers, with more than 2 million new cases being reported in 2020 [2]. Undoubtedly, the currently available breast cancer treatment strategies such as chemotherapy and radiotherapy are associated with a myriad of side effects that severely affect the quality of life of patients [3]. Nevertheless, resistance to anti-cancer drugs and limited specificity towards the target tumor are the challenging issues which could certainly hamper cytostatic therapies, ending with poor outcomes of the breast cancer treatment [4]. Therefore, it is necessary to search for potent alternative therapies, such as phytotherapy, for the treatment of breast cancer.

Phytotherapy involves the use of traditional medicinal plants to target various molecular markers that are altered during cancer. The application of traditional medicinal plants is a promising new anti-cancer strategy to overcome several obstacles in the cancer treatment, including enhancing immune system sensitivity, eliminating inflammation, and preventing metastasis of cancerous cells [5,6]. Recent developments in drug discovery in cancer research have led to the identification and evaluation of a plethora of phytochemicals for their potent anti-cancer efficacy against different cancers. Indeed, more than 100 plant-derived compounds are currently being used as drugs in modern medicine, while many other drugs are simple synthetic modifications of natural phytochemicals [7,8]. Since ancient times, traditional medicinal plants have been used to treat various human ailments including cancer. In fact, a large number of anti-cancer drugs in use today are also derived from plants [9,10].

*Melicope lunu-ankenda* (Evodia lunu-ankenda (Gaertn.) Merr), locally known as “tenggek burung” in Malaysia, is a medicinal plant traditionally used in folk fore medicine to treat various illnesses such as hypertension, diabetes, fever, menstrual disorders, and as an emmenagogue and tonic [11]. It has been reported to possess biological activities including antioxidant, anti-inflammatory, antimicrobial, analgesic, antidiabetic, neuroprotective, and cytostatic activities [10,12,13]. The leaves of *M. lunu-ankenda* have been consumed as a flavoring agent in foods and salads. Natural chromenes extracted from *M. lunu-ankenda* leaves were shown to exhibit antioxidant, immunomodulatory, febrifuge, and analgesic properties [14]. Additionally, several other studies have isolated different biologically active polyphenolic compounds from the leaves of *M. lunu-ankenda* including flavonoids [15], coumarins [16], alkaloids, cinnamic acid derivatives, and benzoic acids derivatives [12]. In our previous study, we have isolated 7-geranyloxycinnamic acid (a derivative of hydroxycinnamic acid) from *M. lunu-ankenda* leaves and demonstrated its neuroprotective effects against H_2_O_2_-induced toxicity using neuroblastoma cell line [17]. Accumulating data suggest the cytotoxic potential of several plants and their polyphenols, including cinnamic acids. In this context, the present study, for the first time, investigated the cytotoxic effects *M. lunu-ankenda* crude leaf extracts against three different cancer cell lines (MCF-7, HT29 and HepG2). Furthermore, we have also evaluated the anti-cancer effects of 7-geranyloxycinnamic acid using breast cancer cell lines (MCF-7 and MDA-MB-231).

## 2. Results

### 2.1. Extraction using Different Solvents

In the present study, we have extracted dried leaf powder of *M. lunu-ankenda* using three different solvents with increasing polarity: petroleum ether, followed by chloroform to methanol. The weight and percentage yield of crude such as petroleum ether, chloroform, and methanol extracts were found to be 61.62, 41.93, 102.16 g and 6.48, 4.37, 10.64%, respectively (Table 1).

#### Isolation and Characterization of 7-Geranyloxycinnamic Acid from the Petroleum Ether Extract of *M. lunu-Ankenda*

**Figure 1:**
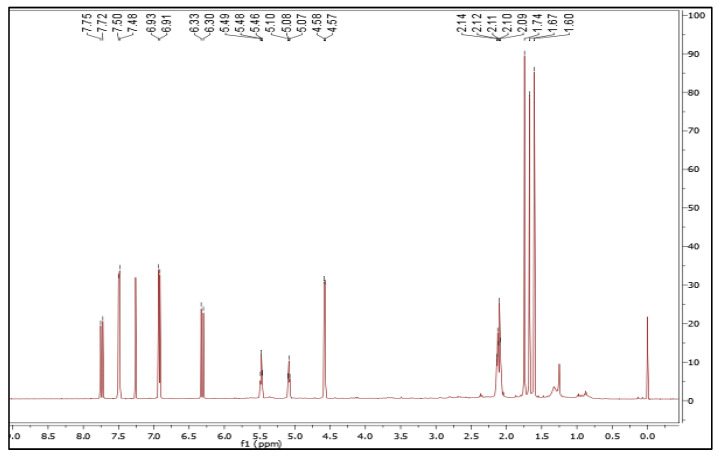
^1^H NMR spectrum of 7-geranyloxycinnamic acid.

**Figure 2:**
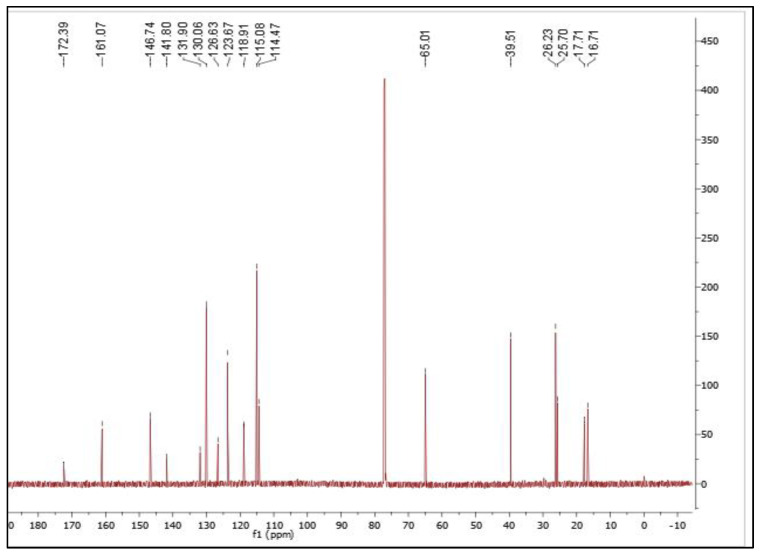
^13^C NMR spectrum of 7-geranyloxycinnamic acid.

The petroleum ether extract (3 g) was subjected to fractionation via column chromatography and a pure compound in the form of white needle-shaped crystals was isolated. The weight and percentage of yield of the isolated white needle-shaped compound was found to be 112 mg and 3.733%, respectively. Nuclear magnetic resonance (NMR) spectroscopy is a powerful tool for full structural elucidation and identification of molecules. Both one-dimensional and two-dimensional NMR experiments are essential for structure identification of chemical compounds. Therefore, in this study, we have performed a combination of 1D and 2D NMR spectroscopy. COSY, HSQC, and HMBC magnetic resonance spectroscopy analysis were performed on the isolated compound (Supplementary Materials). The ^1^H and ^13^C NMR spectra were shown in Figure 1 and Figure 2, respectively, and the ^1^H NMR and ^13^C NMR data for the isolated compound were given in the Table 2.

From the ^1^H NMR data, it was clear that 24 protons are found in the proposed molecular formula of the pure compound. The presence of a methyl group in the molecule is shown by the three singlets, each of which integrated to three protons at δ 1.74, 1.67 and 1.60 (Table 2). The presence of two doublets integrating to one proton each with a coupling constant value of 15 Hz at δ 6.33 and 7.75 (Table 2) clearly indicates the presence of two trans-coupled hydrogens in the isolated compound. The benzene ring with two hydrogen atoms ortho-coupled to one another is confirmed by two further doublets at 6.93 and 7.50 that also integrate to two protons each with a J value of 10 Hz. As a result, two substituents, one of which is a trans-coupled hydrogen moiety, are connected to two additional places on the benzene ring. The H-H COSY spectrum shows coupling between the methylene at δ 4.58 and the methine at δ 5.49, and the two methylenes at δ 2.14 and 2.10 are coupled to the methine at δ 5.10 (Table 2). In contrast, from the HSQC spectrum, the presence of three methylene groups at δ 65.3, 39.8, and 26.5 was found, which gave cross peaks to δ 4.60 and 2.14, respectively.

The ^13^C-NMR spectrum confirms the presence of 19 carbon atoms. The geranyl side chain, which is connected to the oxygen atom at position C-7 of the aromatic ring, has three main carbon atoms attached to three methyl groups at δ 16.7, 17.7 and 25.7. Two carbon atoms, δ 39.5 and 26.2, contain two methylene groups, and at δ 65, a methlenoxy group was present. The spectra also indicated the presence of a carbonyl signal at δ 172.4.

**Figure 3:**
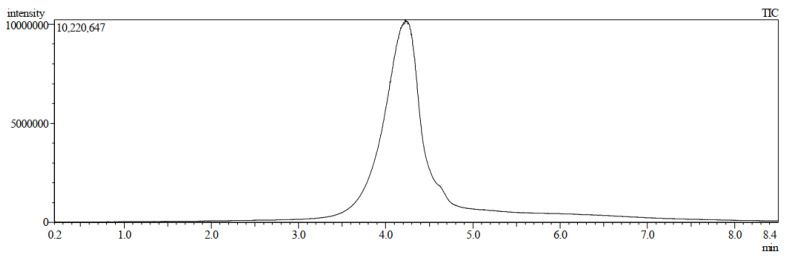
Direct injection probe mass spectrometer (DIP-MS) spectrum of 7-geranyloxycinnamic.

The direct insertion probe−mass spectrometry (DIP−MS) technique has been widely used for the analysis of samples in which samples are directly introduced into the ionization chamber, followed by their vaporization and eventual ionization through electronic impact. The DIP−MS results revealed that the pure compound was reported at 4.240 min with an intensity of about 10,220.647 µV (Figure 3).

**Figure 4:**
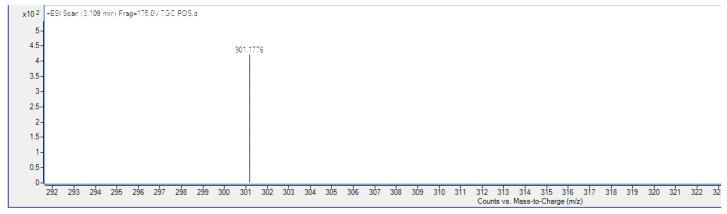
Mass spectrogram (ESI positive mode) of 7-geranyloxycinnamic acid.

Using electron spray ionization (ESI) and liquid chromatography mass spectrometry (LC-MS), which produced a *m*/*z* 301.1776 [M + H] (Figure 4), the identification of the pure compound isolated was further validated and it was similar to the data previously reported by Hashim et al. (2005). From our findings and based on the previously reported reference spectral data (Table 2), the compound was determined to be 7-geranyloxycinnamic acid.

### 2.2. Cytotoxicity of Crude Extracts and 7-Geranyloxycinnamic Acid on Cancer Cell Lines

Following treatment with different concentration of crude extracts for 72 h, the MTT assay was carried out to evaluate the cytotoxic effect against different cell lines and the IC_50_ was calculated. In the current study, the cytotoxic effects of crude extracts (the petroleum ether, chloroform and methanol extracts) were tested against three different cancer cell lines such as HepG2, HT-29, and MCF-7, while the cytotoxicity of 7-geranyloxycinnamic acid was evaluated using two breast cancer cell lines, MCF-7 and MDA-MB-231, as well as a colon cancer cell line (HT-29). The MCF-10a cell line was used as a normal cell line.

#### 2.2.1. Cytotoxicity of Crude Extracts

**Figure 5:**
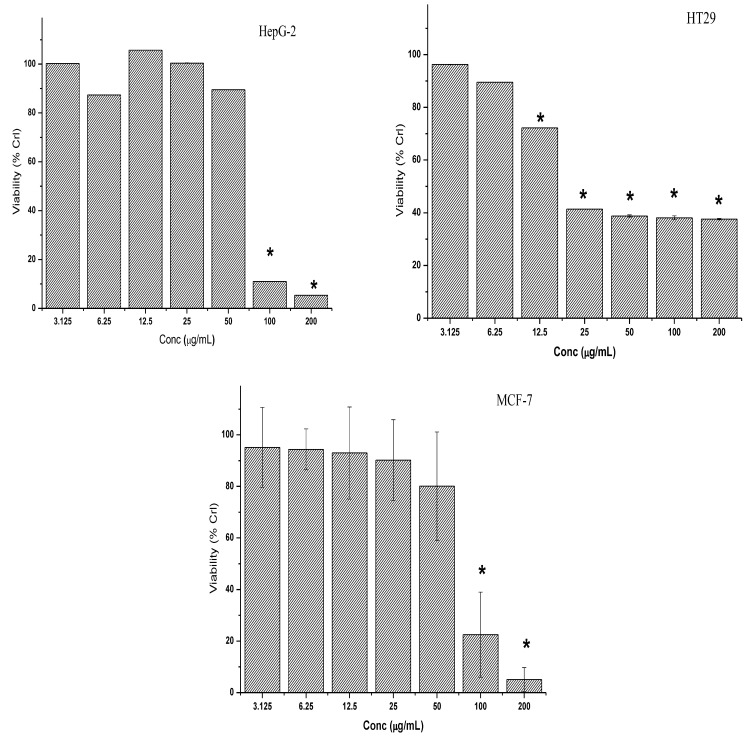
Cytotoxic effect of the petroleum ether extract on HepG2, HT-29 and MCF-7 cancer cell lines. Cells were exposed to increasing concentrations of the crude extracts for 72 h. The results of the three independent tests are shown as the mean ± SD. * Asterisk indicates significant difference at *p* < 0.005.

Petroleum ether extract exposure for 72 h resulted in a slightly dose-dependent increase in the growth inhibition of human colon HT-29 cancer cells (Figure 5). However, the observed cytotoxic effects were found to be significant at all tested concentrations except for 6.25 and 3.125 µg/mL. On the other hand, high concentrations of petroleum ether extract (100 and 200 µg/mL) exerted significant inhibitory effects on the viability of the HepG2 and MCF-7 (Figure 5) cell lines. The viability of the HepG2 and MCF-7 cell lines was found to be 11.0% and 22.5%, respectively, after 72 h of treatment with petroleum ether extract at 100 µg/mL when compared to untreated control cells. When compared to other concentrations examined, the capacity of petroleum ether extract to promote growth inhibition of the MCF-7 and HepG2 cells was highest (about 95% of control) after 72 h exposure to a concentration of 200 g/mL.

**Figure 6:**
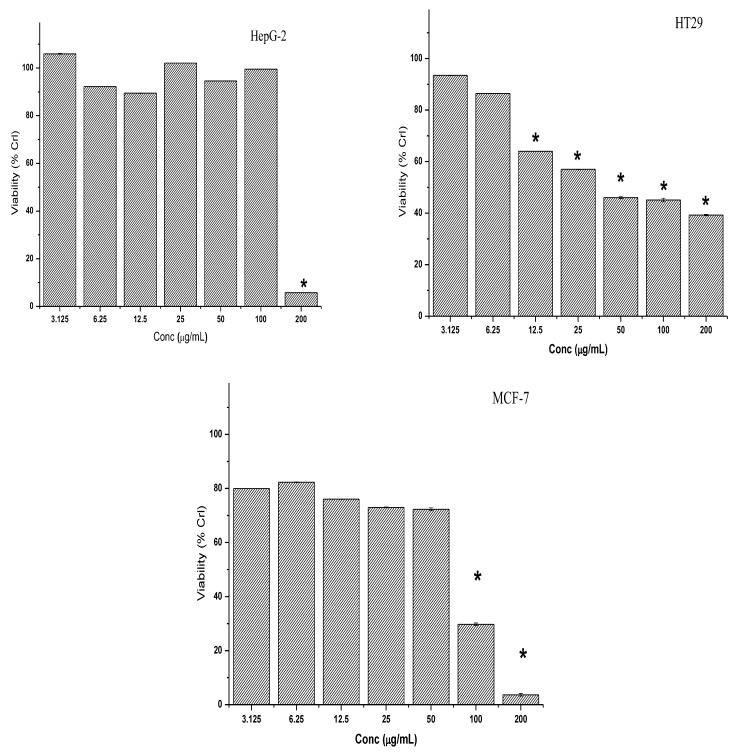
Cytotoxic effect of the chloroform extract on HepG2, HT-29 and MCF-7 cancer cell lines. Cells were exposed to increasing concentrations of the crude extracts for 72 h. The results of the three independent tests are shown as the mean ± SD. * Asterisk indicates significant difference at *p* < 0.005.

Similar to the petroleum ether extract, treatment with chloroform extract for 72 h caused a dose-dependent inhibitory effect on the percentage viability of HT-29 cells and the observed cytotoxic effect was significant for all tested concentrations except at 3.125 and 6.25 µg/mL (Figure 6). In contrast, all tested concentrations of chloroform extract (except 100 and 200 µg/mL) did not exert any significant inhibitory effect on the percentage viability of the MCF-7 and HepG2 cell lines (Figure 6).

**Figure 7:**
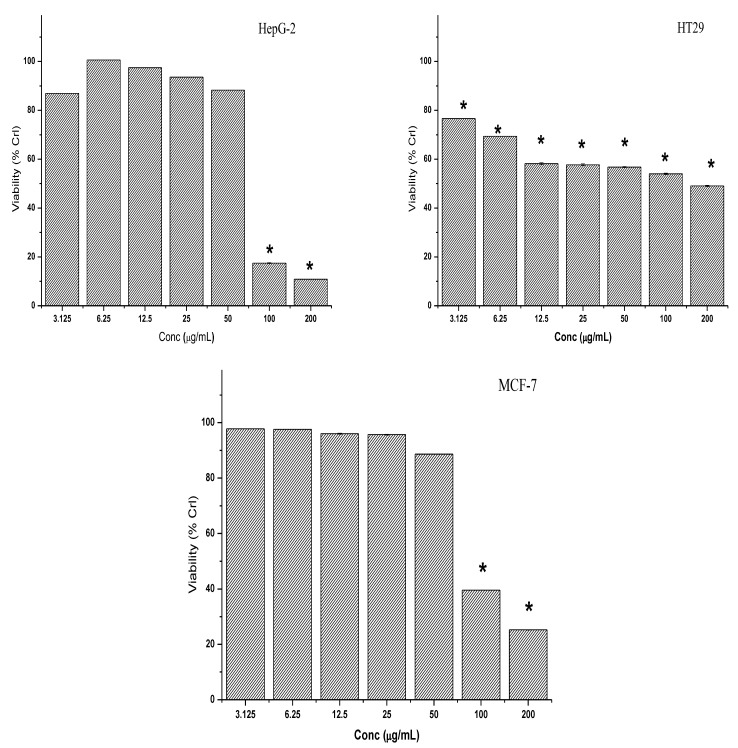
Cytotoxicity effect of the methanol extract on HepG2, HT-29 and MCF-7 cancer cell lines. Cells were exposed to increasing concentrations of the crude extracts for 72 h. The results of the three independent tests are shown as the mean ± SD. * Asterisk indicates significant difference at *p* < 0.005.

In this study, it was also observed that the cytotoxic effects of methanolic extract were similar to those of the petroleum ether extract. Treatment with different concentrations of methanolic extract for 72 h caused a dose-dependent decrease in the viability of HT-29 cells and the cytotoxic effect was found to be significant at all tested concentrations (Figure 7). However, only the higher concentrations of methanol extract could exhibit significant cytotoxic effects against both the HepG2 and MCF-7 cell lines (Figure 7).

The half maximum inhibitory concentrations (IC_50_) were measured for all the tested extracts against each of the different cell lines and the IC_50_ values calculated. The IC_50_ values of petroleum ether extract were found to be low for all tested cell lines when compared to those of the chloroform and methanol extracts (Table 3).

#### 2.2.2. Cytotoxic Effect of 7-Geranyloxycinnamic Acid and 5-Fluorouracil

**Figure 8:**
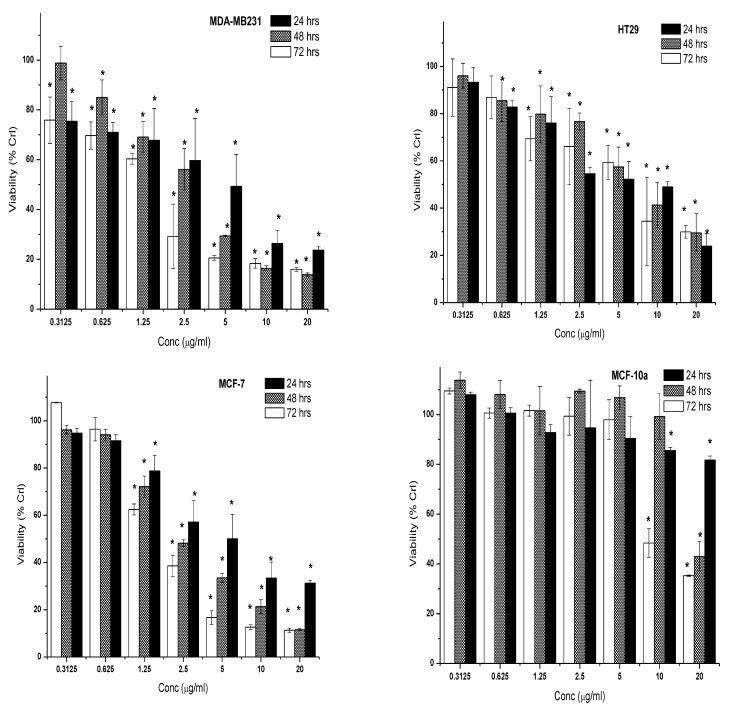
Cytotoxic effect of the 7-geranyloxycinnamic acid on cancer cell lines (MDA MB 231, HT29, MCF-7) and normal cell line (MCF-10a). Cells were incubated with increasing concentrations of the compound in DMEM culture media for 24, 48, and 72 h. Results are expressed as the mean ± SD of three replicates in three independent experiments. * Asterisk indicates significant difference at *p* < 0.005.

The changes in proliferation of three cancer cells (MCF-7, MDA-MB231, and HT29) and normal cell line (MCF-10a) after 24, 48, and 72 h treatment with 7-geranyloxycinnamic acid (at concentrations ranging from 0.3125 to 20.0 µg/mL) are shown in the Figure 8. All tested concentrations of 7-geranyloxycinnamic acid showed a significant cytotoxic effect against the MDA-MBA231 cell line, and the effect was both time dependent as well as dose dependent. In contrast to the untreated control, the time-dependent reduction in cancer cell proliferation was mostly induced by the rising doses of 7-geranyloxycinnamic acid from 1.25 to 20.0 g/mL. When compared to untreated control cells, the MDA-MB-231 cell viability was reduced the most (86.17%) after 48 h of incubation with 7-geranyloxycinnamic acid at a concentration of 20 g/mL (Figure 8).

Except at 0.3125 µg/mL, 7-geranyloxycinnamic acid exerted a time-dependent as well dose-dependent cytotoxicity effect on HT29 cancer cells at a concentration range of 1.25 to 20 µg/mL. When compared to control, the growth inhibitory effect of high concentration (20 µg/mL) of 7-geranyloxycinnamic acid on HT29 cell line was less dramatic than that of MDA-MB231 (Figure 8).

No significant difference (*p* ≥ 0.05) in MCF-7 cell viability was found after 24, 48 and 72-h treatment with 7-geranyloxycinnamic acid at 0.3125 and 0.625 µg/mL compared to control. However, 7-geranyloxycinnamic acid showed a significant decline in percentage viability with time at a concentration range of 1.25 to 20.0 µg/mL (Figure 8). Exposure to a high concentration of 7-geranyloxycinnamic acid for 72 h showed higher cytotoxicity effects on MCF-7 with a lowest percentage viability (11.33%) than on MDA-MB231 (Figure 8). In contrast, 7-geranyloxycinnamic acid at different concentrations, 0.3125, 0.625, 1.25, 2.5 and 5.0 µg/mL, did not exhibit a significant reduction in the viability of MCF-10a cells at all time points (i.e., 24, 48 and 72 h of incubation). Only the high concentration (20 µg/mL) showed a time-dependent yet significant decrease in the percentage viability of MCF-10a cell line by 18.23%, 56.92 and 64.71% after 24, 48 and 72 h of incubation, respectively, when compared to untreated control cells (Figure 8).

The half maximum inhibitory concentrations (IC_50_) were measured for 7-geranyloxycinnamic acid against each of the different cell lines at each of the three different incubation time points. The IC_50_ values of 7-geranyloxycinnamic acid were found to be lowest at all treatment time points against both breast cancer cell lines compared to HT-29 and normal cell lines (Table 4).

**Figure 9:**
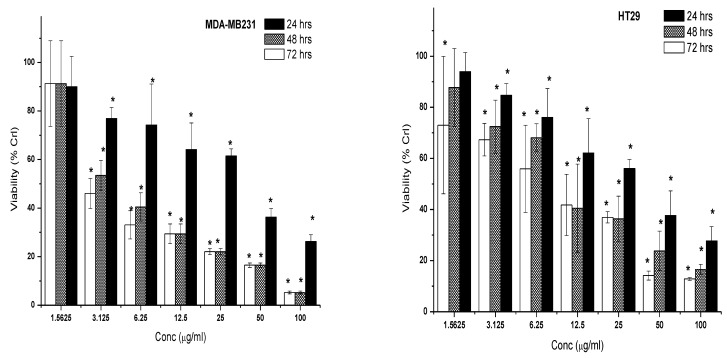

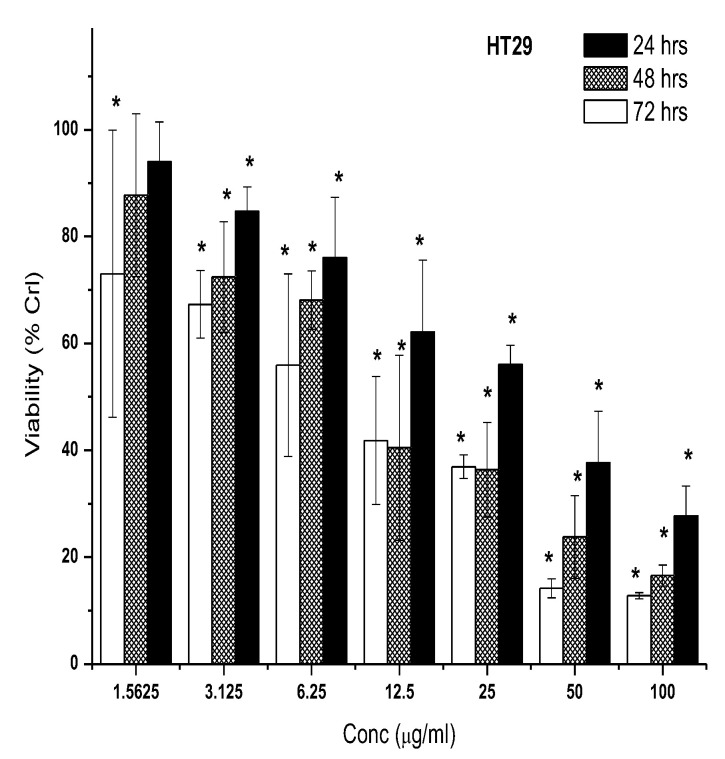
Cytotoxic effect of the 5-fluorouracil on MDA-MB-231, MCF-7, and HT-29 cancer cell lines. Cells were incubated with increasing concentrations of the 5-fluorouracil in DMEM culture media for 24, 48 and 72 h. Results are expressed as the mean ± SD of three replication in three independent experiments. * Asterisk indicates significant difference at *p* < 0.005.

A cytostatic anti-tumor drug for systemic usage is 5-fluorouracil. After 24 h of treatment, it was observed that the percentage of viability of 5-fluorouracil-treated MDA-MB-231 cells at concentrations of 3.125, 12.5, 50.0, and 100.0 g/mL was 23.08%, 35.86%, 63.69%, and 73.73%, respectively, compared to control (Figure 9). After 24 h of exposure, increasing the concentration of 5-fluorouracil from 3.125 to 6.25 g/mL and from 12.5 to 25.0 g/mL did not significantly limit proliferation of MDA-MB231 cells (*p* > 0.05). However, increasing the duration of exposure time to 5-fluorouracil from 24 to 48 h had a substantial anti-proliferative effect (Figure 9). No significant change (*p* > 0.05) in the proliferation of MDA-MB231 cells was noted after 72 h treatment with 5-fluorouracil compared to cells treated with the same concentration for 48 h.

With an increase in dosage and exposure duration, 5-fluorouracil gradually suppressed the proliferation of HT-29 cells. Upon treatment of HT29 cells for 24 h with 5-fluorouracil, their proliferative rate was significantly reduced by 26.97%, 32.69%, 44.06%, 58.17%, 63.08%, 85.83%, and 87.20%, respectively, at doses of 1.536, 3.125, 6.25, 12.5, 25.0, 50.0, and 100 g/mL (Figure 9).

The percentage viability of MCF-7 cells significantly reduced in a concentration- and time-dependent manner. When compared to untreated control cells, the MCF-7 cancer cell line’s vitality significantly decreased to 52.25%, 36.35%, and 9.58% following 24, 48, and 72 h of treatment with 5-fluorouracil at a concentration of 25.0 g/mL (Figure 9). Finally, the half maximum inhibitory concentrations (IC_50_) were measured for 5-fluorouracil against each of the different cell lines and the IC_50_ values calculated were given in the Table 4. With respect to selectivity index (SI), 7-geranyloxycinnamic acid recorded the SI value of (SI > 2) as shown in Table 5.

### 2.3. Morphological Changes in Breast Cancer Cell Lines after Treatment with IC_50_ Concentration of 7-Geranyloxycinnamic Acid

#### 2.3.1. Morphological Changes in MCF-7 Cell Line

From the cytotoxic studies, it is evident that even the low concentrations of 7-geranyloxycinnamic acid can exert profound cytotoxic effects compared to the standard drug. For further studies, we have used three different IC_50_ concentrations of 7-geranyloxycinnamic acid determined for three different incubation time points (24, 48, and 72 h).

**Figure 10:**
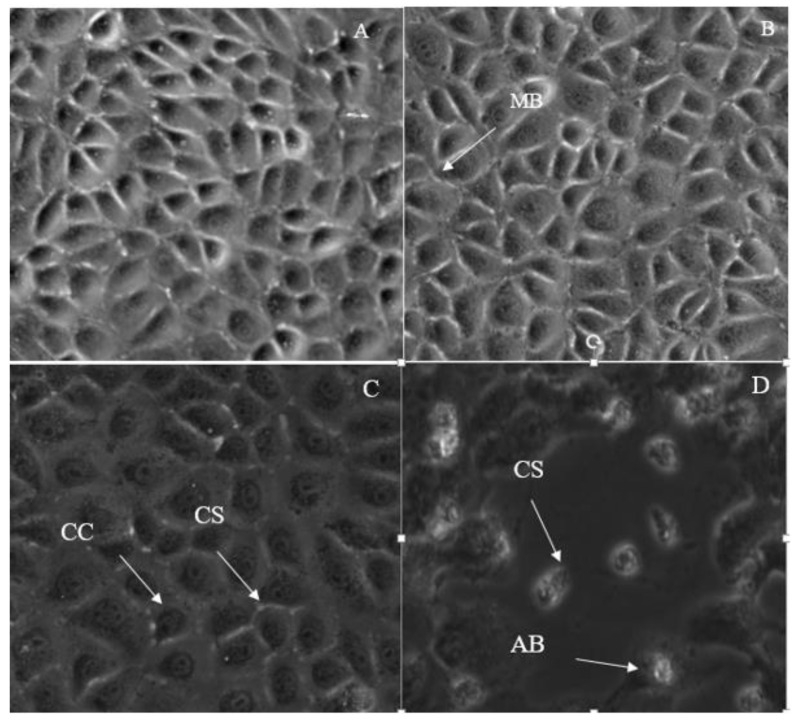
Morphological changes in MCF-7 treated with 7-geranyloxycinnamic acid using phase contrast microscope (200× magnification); (**A**) untreated, (**B**) 24 h, (**C**) 48 h and (**D**) 72 h of incubation. Cell population decreased with the incubation time. MCF-7 cells showed the features of apoptosis such as membrane blebbing (MB), chromatin condensation (CC), cellular shrinkage and rounding (CS) and formation of apoptotic bodies (AB).

In this study, we have used a phase contrast microscope to observe the morphology of MCF-7 cells treated with IC_50_ concentrations of 7-geranyloxycinnamic acid for 24, 48, and 72 h (Figure 10). No significant signs of lysis were detected in MCF-7 cells after 24 h of incubation with IC_50_ concentration of 7-geranyloxycinnamic acid. In contrast, a clear cytotoxic effect of 7-geranyloxycinnamic acid was observed after longer exposure times (48 and 72 h) as evidence by the appearance of small round cells (Figure 10B,C, respectively). Additionally, the lysis of the entire cell population occurred after 72 h of incubation (Figure 10D). The MCF-7 cells were found to be smaller and round after 48 and 72 h of treatment. When compared to untreated control cells, which were intact, with a well-defined nucleus and cytoplasm, the IC_50_ concentration of 7-geranyloxycinnamic acid caused considerable morphological abnormalities in breast cancer MCF-7 cells, as seen in Figure 10. With an increase in the exposure time to IC_50_ concentrations of 7-geranyloxycinnamic acid, the cells are separated from each other. Additionally, a severe cell death and a decline in the total number of MCF-7 cells that displayed a cellular retraction were observed under a microscope.

**Figure 11:**
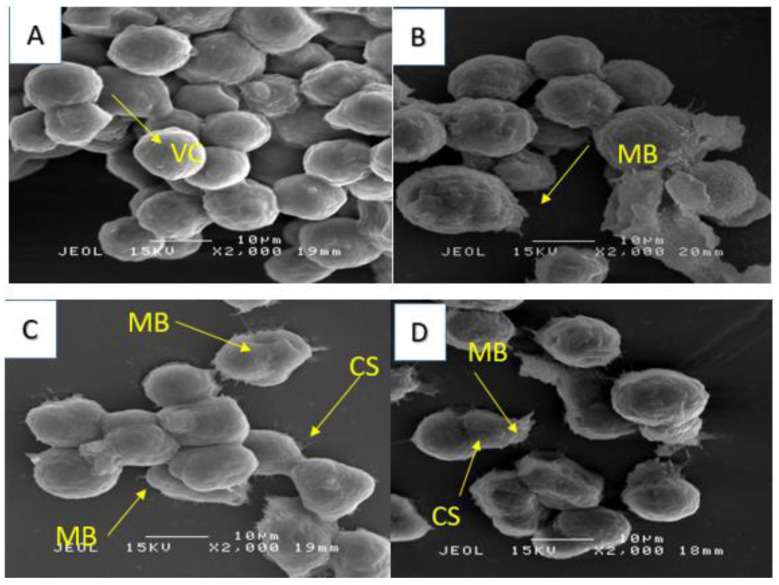
SEM micrograph for MCF-7 cell line. Untreated cells (**A**), treated cells 24 h (**B**), 48 h (**C**) and 72 h, respectively (**D**). (VC) viable cells; MB, membrane blebbing; CS, cytosolic shredding.

**Figure 12:**
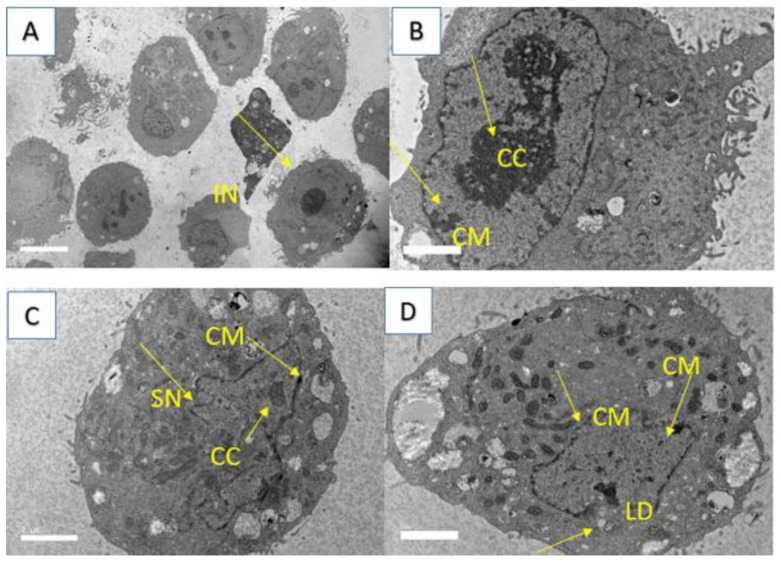
TEM for MCF-7 cell line. (**A**) control, (**B**) treated cells 24 h, (**C**) 48 h and (**D**) 72 h, respectively. IN, Intact nucleus; CC, chromatin condensation; CM, chromatin margination; SN, shrinkage of nuclei; LD, lipid droplet.

**Figure 13:**
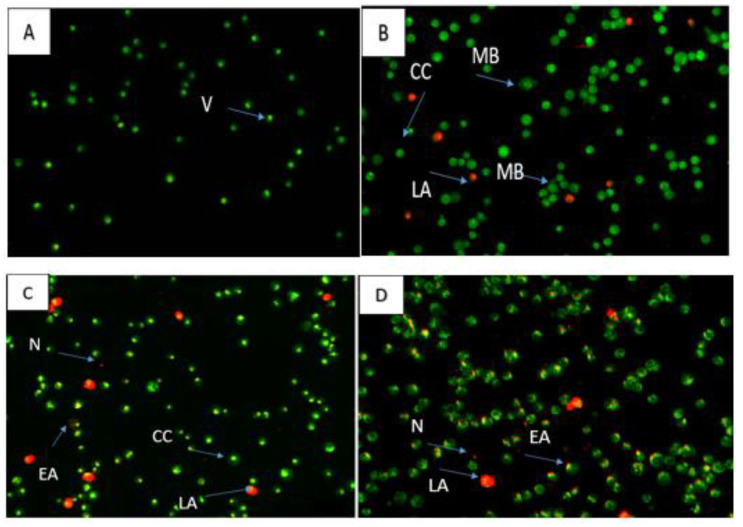
Fluorescent microscopic images of MCF-7 cells stained via AO/PI double staining. Control cell (**A**), cells treated for 24 h (**B**), 48 h (**C**), and 72 h (**D**). Viable cells (V), early apoptosis (EA), chromatin condensation (CC), membrane blebbing (MB), late apoptosis (LA), necrosis (N).

Additionally, using a scanning electron microscope, we observed structures similar to vacuoles in the cytoplasm of the cells (Figure 11), while cytoplasmic fragmentation was observed using a transmission microscope (Figure 12). These alterations became more pronounced with longer exposure times and resembled those induced in the human melanoma cell line (HT-144) after 48 h of incubation with cinnamic acid, a similar pro-apoptotic compound to our compound [18]. Additionally, after 48 and 72 h of incubation with IC_50_ concentrations of 7-geranyloxycinnamic acid, enlarged cytoplasm, presence of blebs and vacuoles, and loss of plasma membrane were observed under both electron microscopes. The integrity of the cell membrane was also harmed by the 7-geranyloxycinnamic acid after 48 and 72 h of incubation, as demonstrated by a transmission electron microscope (Figure 12). Thus, it is clear from the SEM and TEM analyses that 7-geranyloxycinnamic acid treatment of MCF-7 cells at the IC_50_ concentration caused a considerable rise in the cellular retraction over time, along with alterations in cell contours and loss of connections between MCF-7 cells. 7-geranyloxycinnamic acid was found to exert a profound cytotoxic effect only when cells were exposed to 48 and 72 h with IC_50_ concentration and showed substantial morphological changes since the majority of dead MCF-7 cells were at late apoptotic stage and had lost their membrane integrity (red fluorescent color) (Figure 13).

Apoptosis is observed through dual staining with acridine orange and propidium iodide. Propidium iodide and acridine orange designate physically normal cells and apoptotic cells, respectively, by fluorescing from green to yellow and red when bound to DNA and cell membrane, respectively. After 48 and 72 h of treatment with 7-geranyloxycinnamic acid at IC_50_ concentration, viable MCF-7 cancer cells exhibit uniformly distributed green fluorescence (Figure 13), but early apoptotic cells exhibit cell membranes exhibiting a higher yellow fluorescence intensity. Contrarily, long exposure times (72 h) to 7-geranyloxycinnamic acid only cause propidium iodide to stain the nuclei of cells when membrane integrity or permeability is disturbed, which may signify late apoptosis but is typically linked to necrotic processes [19].

#### 2.3.2. Morphological Changes in MDA-MB231 Cell Line

**Figure 14:**
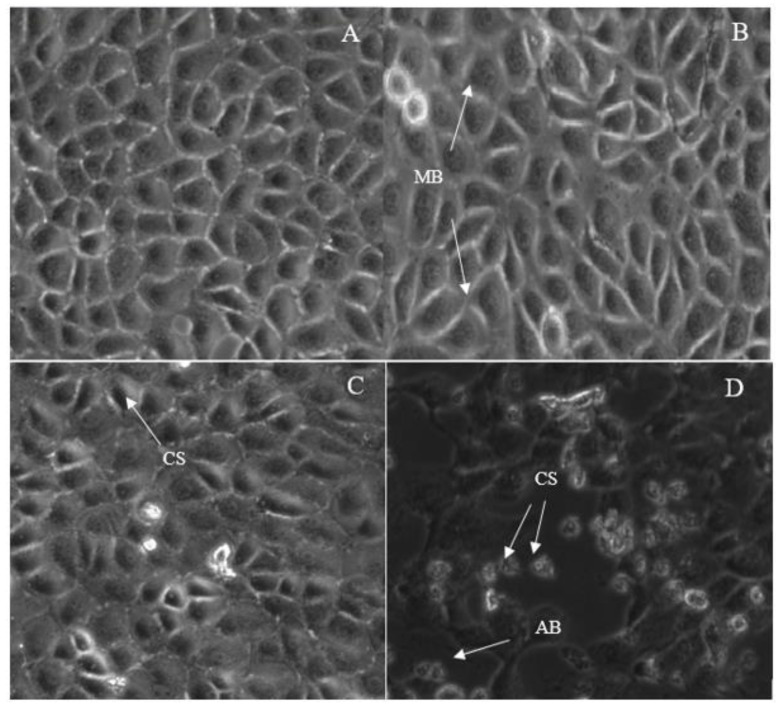
Morphological changes of treated MDA-MB-231 cells observed under phase contrast microscope (200× magnification). Untreated cells (**A**), treated cells 24 h (**B**), 48 h (**C**), and 72 h (**D**). Cell population decreased with the incubation time. The cells showed the features of apoptosis such as membrane blebbing (MB), cellular shrinkage and rounding (CS) and formation of apoptotic bodies (AB).

The phase contrast microscopic examination was also carried out on the MDA-MB-231 cell line treated with IC_50_ concentrations determined for 24, 48 and 72 h of exposure time points (Figure 14). The MDA-MB231 cells showed a clear spherical morphology compared to the untreated control, indicating that cells were highly sensitivity to IC_50_ concentrations of 7-geranyloxycinnamic acid at 48 and 72 h of incubation. Interesting to note is that after 72 h treatment with 7-geranyloxycinnamic acid, the entire cell population died. Similar to MCF-7 cell line, the MDA-MB-231 cells were lysed after 72 h incubation with 7-geranyloxycinnamic acid (Figure 14D).

**Figure 15:**
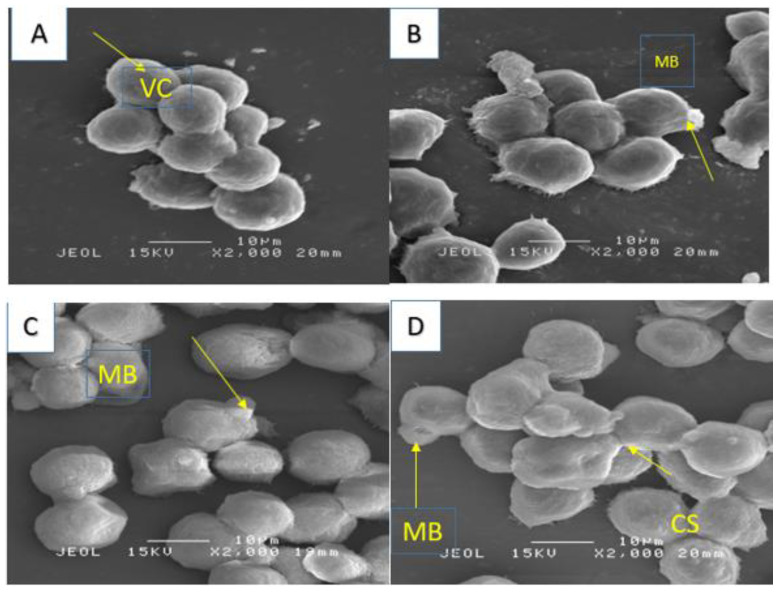
SEM micrograph for MDA-MB-231 cell line. Untreated cells (**A**), treated cells 24 h (**B**), 48 h (**C**) and 72 h, respectively (**D**). (VC) viable cells; MB, membrane blebbing; CS, cytosolic shredding.

**Figure 16:**
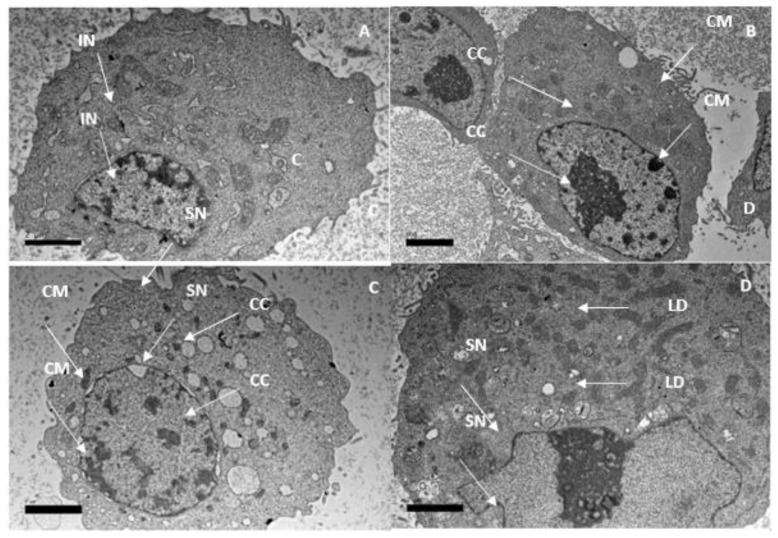
TEM for MDA-MB-231 Cell line. (**A**) control, (**B**) treated cells 24 h, (**C**) 48 h and (**D**) 72 h, respectively. IN, Intact nucleus; CC, chromatin condensation; CM, chromatin margination; SN, shrinkage of nuclei; LD, lipid droplet.

**Figure 17:**
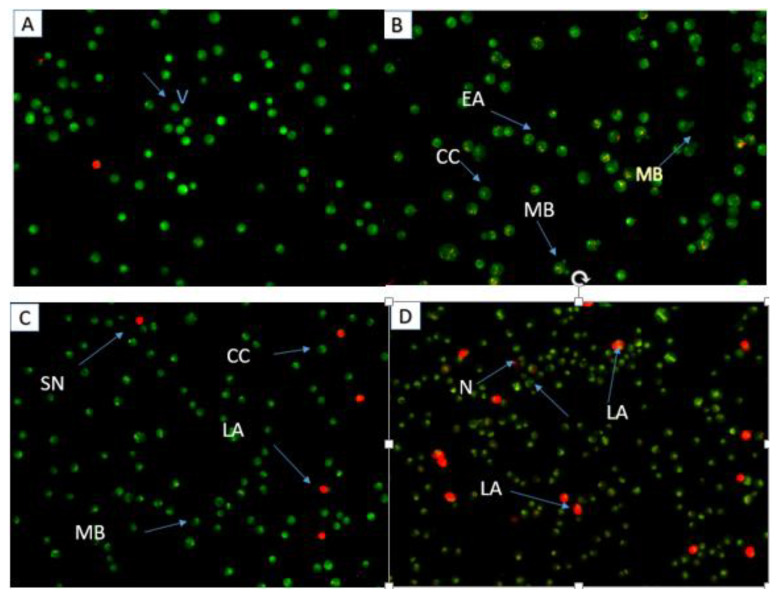
Fluorescent micrograph of MDA-MB-231 cells via (AO/PI). Untreated cells (**A**), treated cells 24 h (**B**), 48 h (**C**), and 72 h (**D**). Viable cells (V), early apoptosis (EA), chromatin condensation (CC), membrane blebbing (MB), late apoptosis (LA), necrosis (N).

In addition, 7-geranyloxycinnamic acid at IC_50_ concentration promoted profound morphological changes in MDA-MB-231 after 24, 48, and 72 h of incubation as evidenced using scanning electron microscopy (Figure 15), transmission electron microscopy (Figure 16), and fluorescent microscopy analyses (Figure 17). From the TEM images, we can clearly see the cell retraction and presence of structures similar to vacuoles inside the cytoplasm, which became denser and more stained with yellow then red color with increasing exposure time, as seen from fluorescent microscopic images. SEM observations revealed changes in cell shape and disruptions at cell junctions that support the separation of nearby cells (Figure 15). Nuclear changes were also observed; as a result, the nucleus got denser and slightly shifted towards periphery (Figure 16). From the TEM morphological studies, it was clearly evident that MDA-MB-231 cells showed apoptotic features following 72 h of treatment with 7-geranyloxycinnamic acid at IC_50_ concentration. In addition, after being exposed to 7-geranyloxycinnamic acid for 48 and 72 h, the MDA-MB-231 cells displayed the presence of blebs and an increase in cytoplasmic size.

The apoptotic potential of the 7-geranyloxycinnamic acid was evaluated using dual staining with acridine orange and propidium iodide in the MDA-MB-231 cancer cell line (Figure 17). After treatment with 7-geranyloxycinnamic acid for 48 and 72 h, MDA-MB-231 cells were also stained with propidium iodide (red color), indicating compromised cell membrane permeability. Untreated cells showed nuclei with uniformly distributed green fluorescence. On the other hand, treatment with 7-geranyloxycinnamic acid at IC_50_ concentration lead to nuclear fragmentation and chromatin condensation in MDA-MB-231 cells. Furthermore, there is a reduction in the number of viable cells compared to the untreated cells, indicating the cytotoxic effect of our compound. Propidium iodide only stains the nucleus of cells that have lost membrane integrity. However, in this study, no propidium-iodide-stained positive MDA-MB-231 cells were observed upon treatment with compound for 24 h (Figure 17B), which indicates that the 7-geranyloxycinnamic acid under study does not induce necrosis in MDA-MB-231 or the dead cells were at early apoptotic stage.

### 2.4. Apoptosis Assessment

**Figure 18:**
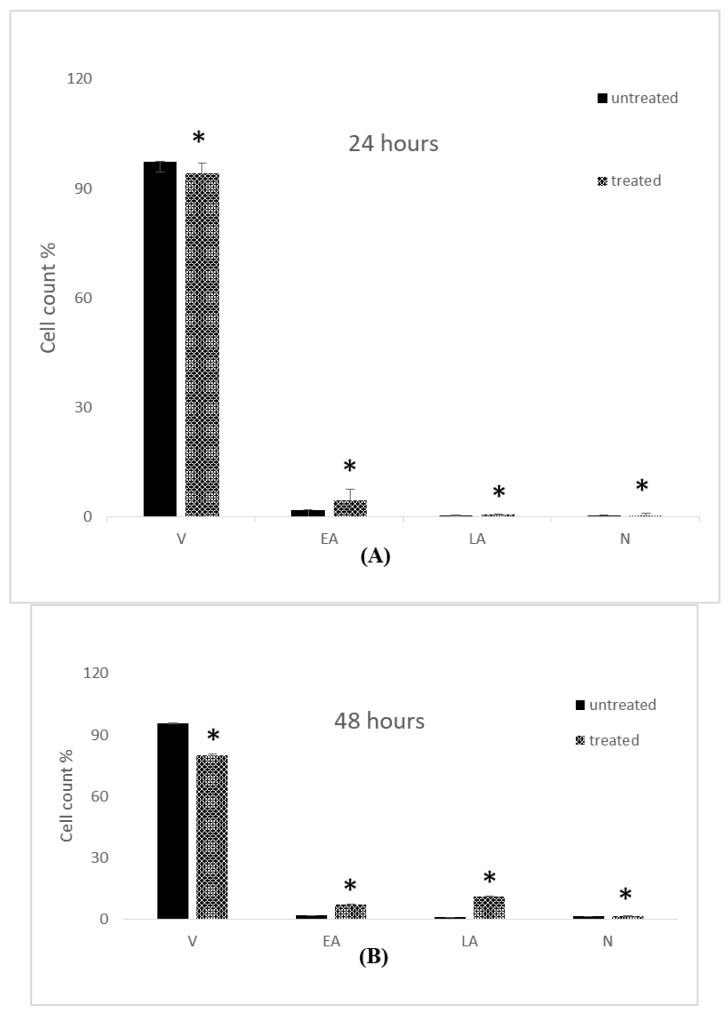

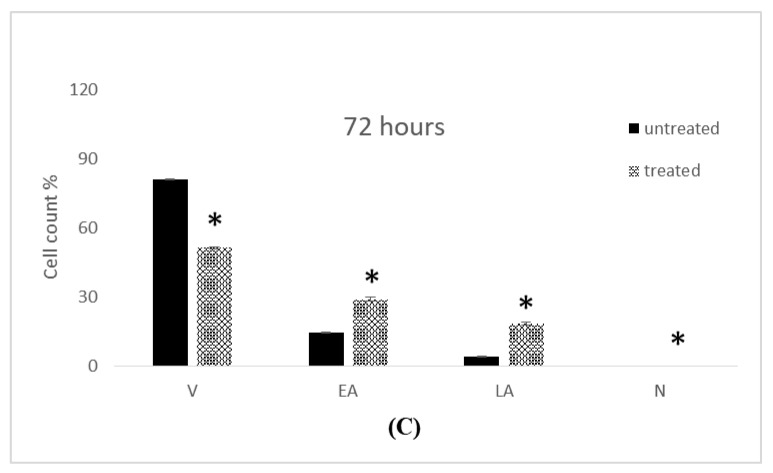
Annexin V-FITC assay of MCF-7 cell line determined using flow cytometry. The percentage of viable, early apoptotic, late apoptotic and necrotic cells of untreated and treated MCF-7 cells. (**A**) 24 h, (**B**) 48 h and (**C**) 72 h. Each data point represents the mean of three independent experiments ± SD. * Significantly different from the control (*p* < 0.05). V: viable; EA: early apoptosis; LA: late apoptosis; N: necrosis.

Flow cytometric analysis was carried out to assess the apoptosis of breast cancer cell lines treated with IC_50_ concentrations of 7-geranyloxycinnamic acid, as shown in the Table 4. Flow cytometric analysis of apoptotic events in MCF-7 cell lines is shown in the Figure 18. It is evident from the figure that treatment with IC_50_ concentrations of 7-geranyloxycinnamic acid resulted in a significant decrease in the percentage of viable MCF-7 cells with an increase in of exposure time. The decrease in percentage viability was found to be 94.2, 80.2 and 51.4% after incubation for 24, 48, and 72 h, respectively. It was clear from the figure that most apoptotic cells are in an early stage; therefore, they are FITC+/PI−. Exposure to 7-geranyloxycinnamic acid resulted in an increase in the percentage of early apoptotic cells with an increase in time of exposure. In comparison to untreated control cells, the increase in the percentage of early apoptotic cells was found to be 250.9, 382.4, and 200% at 24, 48, and 72 h of incubation with 7-geranyloxycinnamic acid (Figure 18). Similarly, a significant increase (*p* < 0.05) in the number of cells in late apoptosis in the presence of the 7-geranyloxycinnamic acid with an increase in time of exposure was also observed (Figure 18).

**Figure 19:**
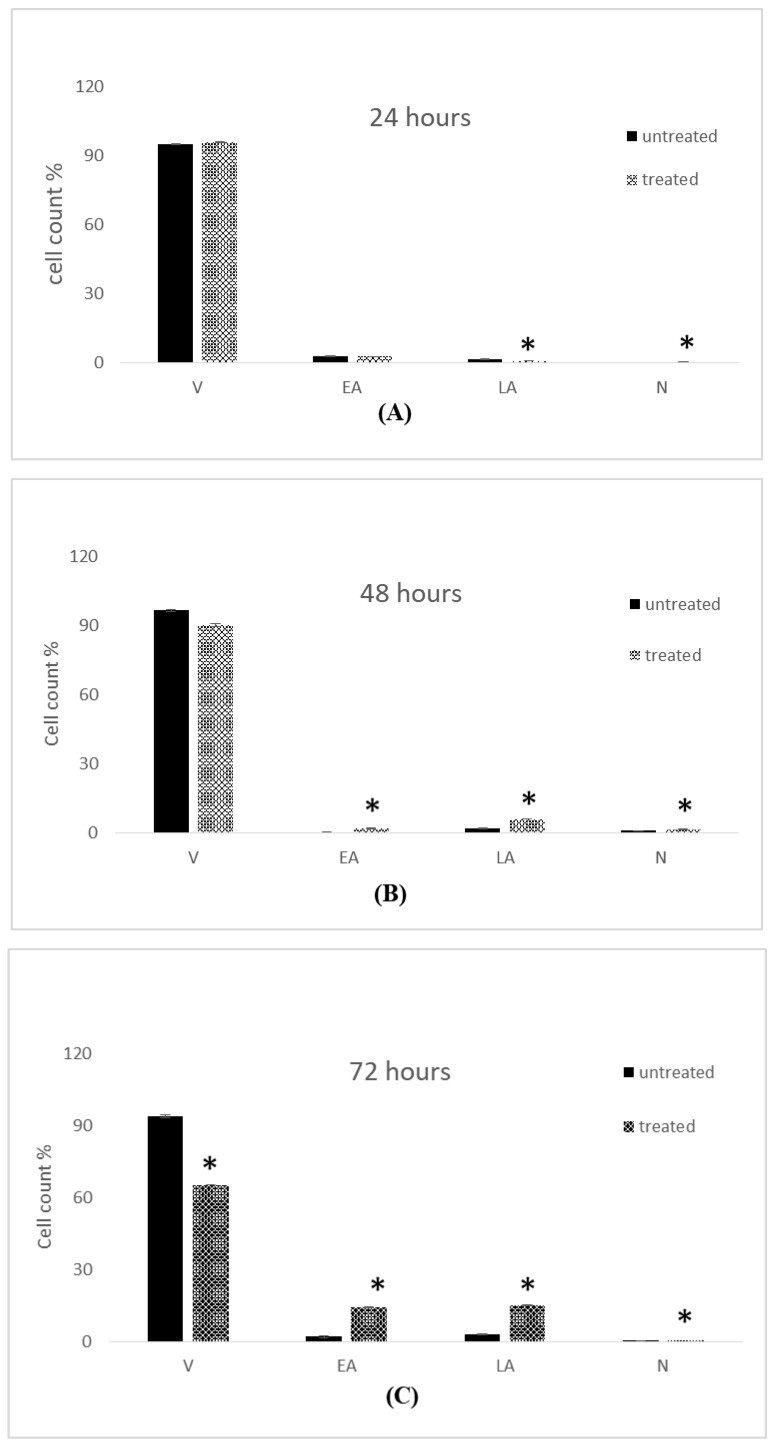
Annexin V-FITC assay of MDA-MB-231 cell line determined using flow cytometry. The percentage of viable, early apoptotic, late apoptotic and necrotic cells of untreated and treated MCF-7 cells. (**A**) 24 h, (**B**) 48 h and (**C**) 72 h. Each data point represents the mean of three independent experiments ± SD. * Significantly different from the control (*p* < 0.05). V: viable; EA: early apoptosis; LA: late apoptosis; N: necrosis.

Figure 19 shows the effect of IC_50_ doses of 7-geranyloxycinnamic acid on the MDA-MB-231 cell line’s apoptotic cell death at 4.85, 3.07, and 1.67 g/mL. The percentage of viable MDA-MB-231 cells (FITC-/IP-) reduced dramatically (*p* < 0.05) from 95.43% (compared to control) to 67.63% after 72 h of incubation with 7-geranyloxycinnamic acid. The proportion of viable MDA-MB-231 cells (FITC−/IP−) did not change significantly (*p* ≥ 0.05) after 24 and 48 h of treatment with the IC_50_ values of 7-geranyloxycinnamic acid at 5.368 and 1.732 g/mL, respectively, in comparison to control. After 48 and 72 h of treatment with the corresponding IC_50_ doses of 7-geranyloxycinnamic acid, the percentage of cells in an early stage of apoptosis increased considerably (*p* ≤ 0.05) from 0.37 and 2.30% to 2.13 and 15.83% (Figure 19). Following 24 h of exposure to 7-geranyloxycinnamic acid at a concentration of 5.368 g/mL, there was no noticeable difference (*p* ≥ 0.05) in MDA-MB231 cells at early stage of apoptosis (Annexin V+/PI) compared to control cells (Figure 19A). After 48 and 72 h of treatment with 7-geranyloxycinnamic acid at concentrations of 3.975 and 1.732 g/mL, respectively, the percentage of late apoptotic cells (Annexin V+/PI+) increased considerably (*p* < 0.05) from 1.93 and 2.23% to 6.20 and 16.37%. After 24 h of treatment with 7-geranyloxycinnamic acid at a concentration of 5.368 g/mL, the cells at the late stage of apoptosis considerably (*p* < 0.05) dropped from 1.80% to 1.00%. Similar to MCF-7 cells, a small proportion of MDA-MB-231 cells succumbed to necrosis and were labeled (FITC−/PI+) after being incubated with 7-geranyloxycinnamic acid for 24, 48, and 72 h at doses of 5.368, 3.975, and 1.732 g/mL, respectively (Figure 19B,C).

In order to confirm the fluorescence microscopic results, we have used flow cytometry, a technique with greater specificity and sensitivity than the morphological studies [20,21,22]. Our flow cytometric data together with fluorescence microscopic, SEM and TEM analysis studies confirm that 7-geranyloxycinnamic acid can promote the induction of the apoptotic process in the breast cancer MDA-MB-231 and MCF-7 cell lines.

### 2.5. Cell Cycle Analysis

We have examined the influence of IC_50_ concentrations of 7-geranyloxycinnamic acid on the cell cycle events in MCF-7 and MDA-MB231 cell lines. Cancer cell lines were incubated with IC_50_ concentrations of 7-geranyloxycinnamic acid at the indicated times points following which cells were stained and analyzed using flow cytometer.

#### 2.5.1. Cell Cycle Analysis of MCF-7 Cells

**Figure 20:**
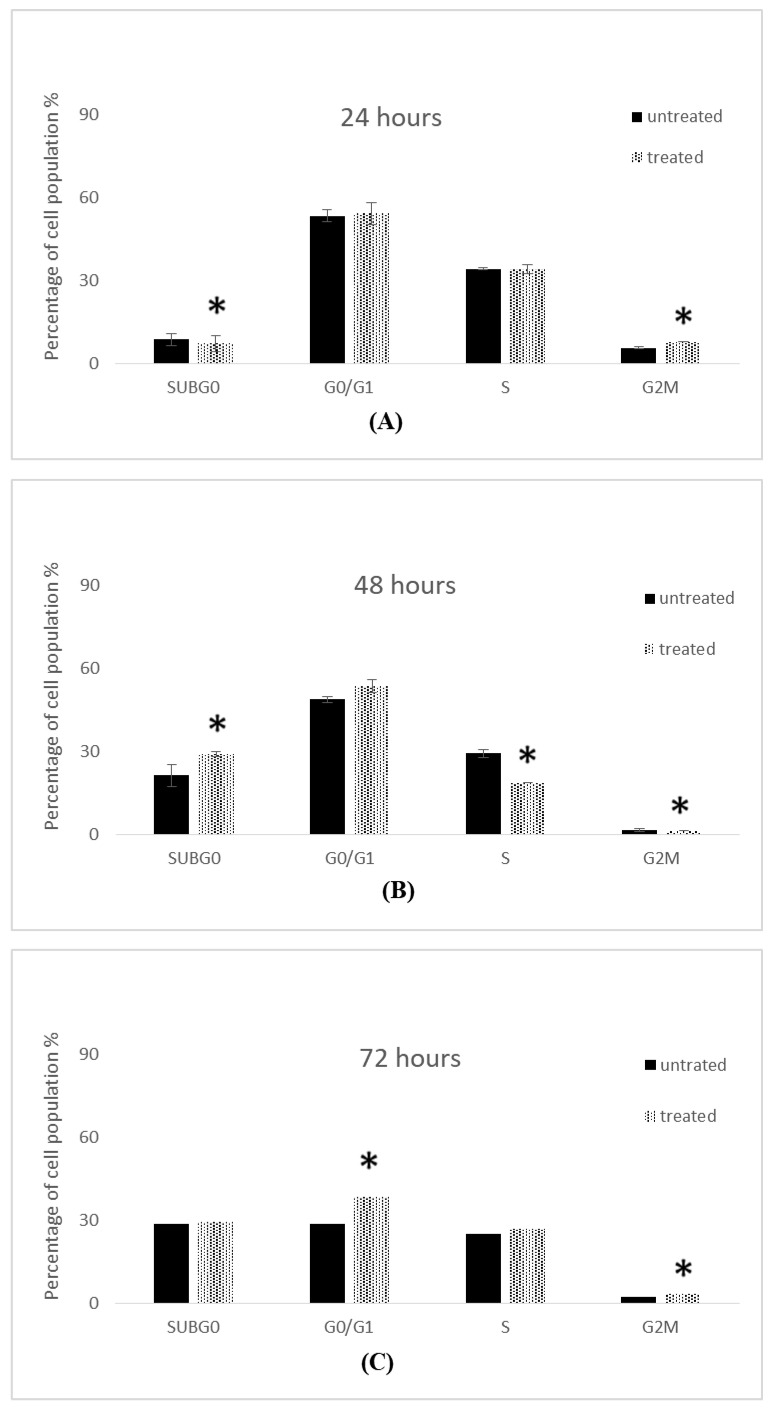
Cell cycle profile of MCF-7 cells treated with 7-geranylcinnamic acid. Each data point represents the mean of three independent experiments ± SD. * Significantly different from the control (*p* < 0.05). (**A**) 24 h, (**B**) 48 h and (**C**) 72 h of incubation.

The influence of IC_50_ concentrations of 7-geranyloxycinnamic acid at 4.96, 2.41 and 1.81 µg/mL (corresponding to 24, 48, and 72 h) on the cell cycle of MCF-7 cells is shown in Figure 20. The proportion of cells in G0/G1 increased slightly in 7-geranyloxycinnamic acid-treated cells after 24 h incubation. In contrast, the proportion of cells in G0/G1 increased notably from 48.75 ± 1.09% (with control cells) to 53.55 ± 2.48%. The population of cells in G0/G1 phase increased significantly (*p* < 0.05) from 28.90 ± 0.69% to 38.65 ± 1.33% after 72 h of incubation with 7-geranyloxycinnamic acid at a concentration of 1.81 µg/mL. However, following 48 h of exposure to 2.41 g/mL of 7-geranyloxycinnamic acid, the number of cells in sub-Gl phase increased significantly (*p* < 0.05), from 21.25 ± 4.09% to 29.15 ± 0.87% (Figure 20B). After 24 and 72 h of exposure to 7-geranyloxycinnamic acid at concentrations of 4.96 and 1.81 g/mL, respectively, no significant alterations (*p* ≥ 0.05) in sub-Gl phase were seen in the MCF-7 cells (Figure 20A,C).

The proportion of cells in S-phase declined remarkably (*p* < 0.05), from 29.35 ± 1.44% to 18.7 ± 0.12%, after 48 h treatment with 2.41 µg/mL of 7-geranyloxycinnamic acid (Figure 20B). After 72 h of exposure to 1.81 g/mL of 7-geranyloxycinnamic acid, there was a small increase in the population of MCF-7 cells that were in the S-phase, but 24 h of exposure to 4.96 g/mL showed no change in the percentage of cells that were in the S-phase. Additionally, following 24 and 48 h of incubation with 4.96 and 1.81 g/mL of 7-geranyloxycinnamic acid, respectively, the cell populations at M/G2 phase significantly (*p* < 0.05) rose from 5.70 ± 0.35% and 2.45 ± 0.87% with untreated MCF-7 cells to 7.95 ± 0.17% and 3.45 ± 1.33%.

#### 2.5.2. Cell Cycle Analysis of MDA-MB-231 Cells

**Figure 21:**
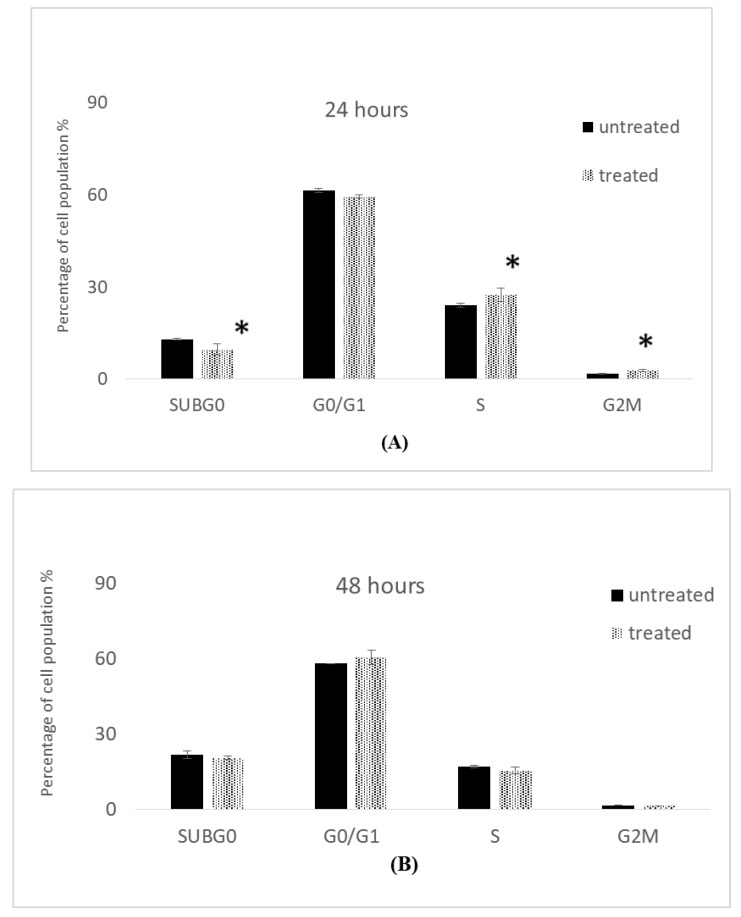

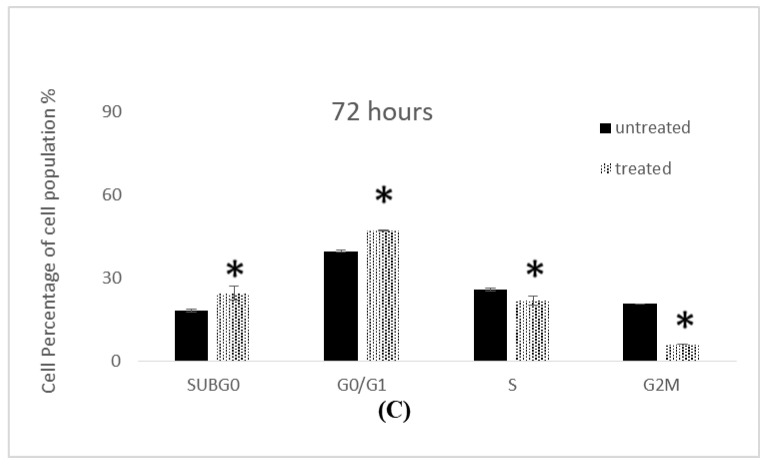
Cell cycle profile of MDA-MB-231 cells treated with 7-geranyloxycinnamic acid. Each data point represents the mean of three independent experiments ± SD. * Significantly different from the control (*p* < 0.05). (**A**) 24 h, (**B**) 48 h and (**C**) 72 h of incubation.

Figure 21 shows the effect of 7-geranyloxycinnamic acid on the cell cycle analysis of breast cancer MDA-MB-231 cells at IC_50_ concentrations of 4.85, 3.07, and 1.67 g/mL. The percentage of cells in the sub-Gl phase increased considerably from 18.15 ± 0.52% to 24.00 ± 0.45% after 72 h of treatment with 7-geranyloxycinnamic acid at 1.67 g/mL. In contrast, the number of cells in the sub-Gl phase increased significantly (*p* < 0.05) from 13.05 ± 0.17% to 9.65 ± 1.79% after 24 h of treatment with 7-geranyloxycinnamic acid at a concentration of 4.85 g/mL, while MDA-MB-231 cells showed no significant change in the sub-Gl phase after 48 h of incubation with a concentration of 3.07 g/mL when compared to control (Figure 21B).

The proportion of cells in the G0/G1 phase increased significantly (*p* < 0.05) from 39.65 ± 0.40% to 47.15 ± 0.06% after 72 h incubation with 7-geranyloxycinnamic acid at a concentration of 1.67 µg/mL. The percentage of cells in G0/G1 phase dropped (*p* ≥ 0.05) from 61.55 ± 0.64% to 59.50 ± 0.46% after 24 h of incubation with 7-geranyloxycinnamic acid at a concentration of 4.85 µg/mL. No noticeable increase (*p* ≥ 0.05) in the G0/G1 phase was seen for MDA-MB231 cells treated for 48 h with 7-geranyloxycinnamic acid at a concentration of 3.07 µg/mL when compared to the control cells (Figure 21B).

Additionally, following 24 h of incubation with 7-geranyloxycinnamic acid at a concentration of 4.85 g/mL, the percentage of cells in the S-phase increased significantly (*p* < 0.05) from 24.10 ± 0.58% to 27.55 ± 2.25%. (Figure 21A). After 48 h of treatment with 3.07 µg/mL, the proportion of cells in S-phase declined from 17.05 ± 0.52% to 15.40 ± 1.27% (Figure 21B). After 72 h of incubation with 7-geranyloxycinnamic acid at a concentration of 1.67 g/mL, a substantial decrease (*p* < 0.05) was seen in the number of cells in the S-phase, from 25.90 ± 0.57% to 21.75 ± 1.67%.

Additionally, following 48 h of exposure to 7-geranyloxycinnamic acid at an IC_50_ value of 3.07 g/mL, the percentage of cells in the M/G2 phase reduced negligibly from 1.60 ± 0.00% to 1.50 ± 0.00%. The proportion of cells in the M/G2 phase reduced substantially (*p* < 0.05) from 20.00 ± 0.00% to 6.10 ± 0.00% after 72 h of treatment with 1.67 µg/mL of 7-geranyloxycinnamic acid (Figure 21C). After 24 h of treatment with 7-geranyloxycinnamic acid at an IC_50_ concentration of 4.85 g/mL, the percentage of MDA-MB-231 cells significantly increased (*p* < 0.05) in the M/G2 phase.

### 2.6. Induction of Intracellular Reactive Oxygen Species (ROS) by 7-Geranyloxycinnamic Acid

**Figure 22:**
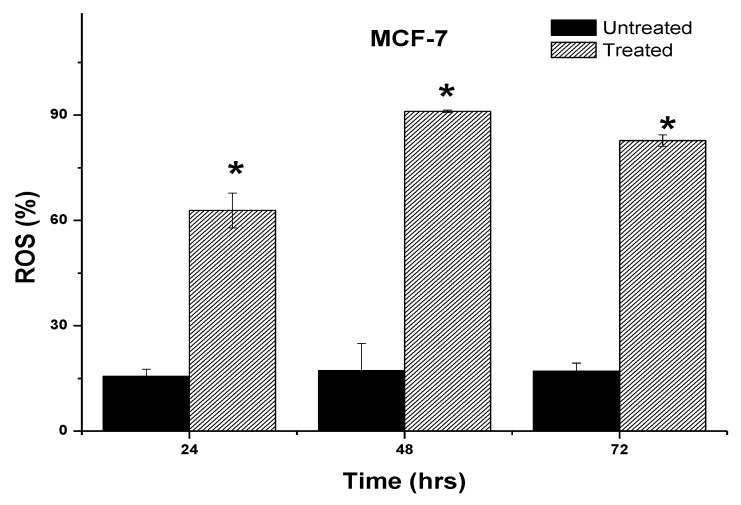
Percentage of reactive oxygen species in MCF-7 cells after treatment with 7-geranyloxycinnamic acid for 24, 48 and 72 h. Error bar represents mean ± standard deviation. * Significantly (*p* ˂ 0.05) different from control.

To quantify the fluorescence intensity of the H_2_DCF-DA dye in the cancer cell lines, the cells were treated with IC_50_ concentrations of the pure compound and incubated for the time points indicated. Our findings show that two breast cancer cell lines exposed to 7-geranyloxycinnamic acid had alterations in fluorescence intensity compared to untreated control cells. Treatment with 7-geranyloxycinnamic acid at a concentration of 4.96 µg/mL for 24 h resulted in a significant increase in fluorescent intensity (402.9%), indicating the accumulation of ROS in MCF-7 cells line when compared to the untreated control cells (Figure 22). Accumulation of ROS in MCF-7 cells was more prominent (529.4%) after treatment with 7-geranyloxycinnamic acid at a concentration of 2.41 µg/mL for 48 h when compared to the untreated control. No significant (*p* > 0.05) accumulation of ROS was observed after 72 h of exposure to 7-geranyloxycinnamic acid at an IC_50_ concentration when compared to 48 h of incubation.

**Figure 23:**
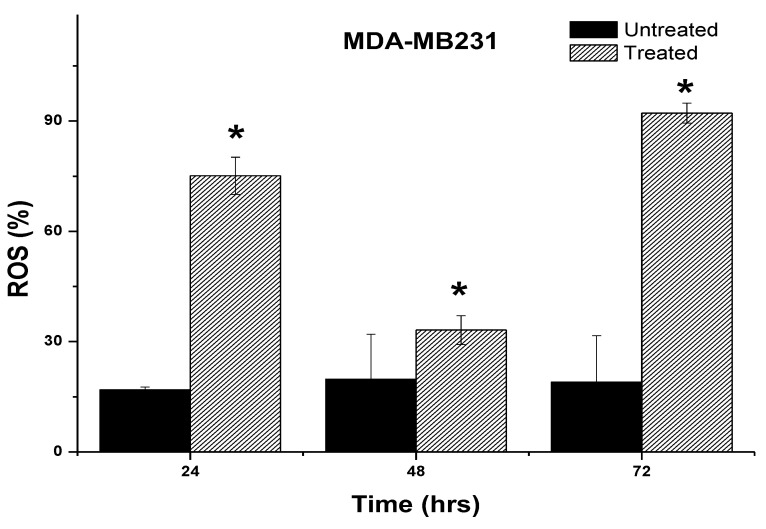
Reactive oxygen species for MDA-MB-231 cells after treatment with 7-geranyloxycinnamic acid at 24, 48 and 72 h. Error bar represents mean ± standard deviation. * Significantly (*p* ˂ 0.05) different from control.

The intracellular ROS profile of MDA-MB-231 treated with 7-geranyloxycinnamic acid for different time points is depicted in Figure 23. A significant increase in the intracellular ROS levels was observed in MDA-MB-231 cell lines upon treatment with IC_50_ concentrations of 7-geranyloxycinnamic acid when compared to the untreated cells and this effect was found to be time dependent. The ROS levels were found to be significantly (*p* < 0.05) increased, from 16.90 ± 0.77% and 19.20 ± 12.62% to 75.13 ± 5.06% and 92.19 ± 2.73%, respectively, after 24 and 72 h of incubation with 7-geranyloxycinnamic acid. From this, it can be suggested that 7-geranyloxycinnamic acid can induce the accumulation of intracellular ROS levels, thereby exerting cytotoxic effects against the breast cancer cell lines tested.

### 2.7. Determination of Caspases-3/7, -8 and -9 Activity Induced by 7-Geranyloxycinnamic Acid

**Figure 24:**
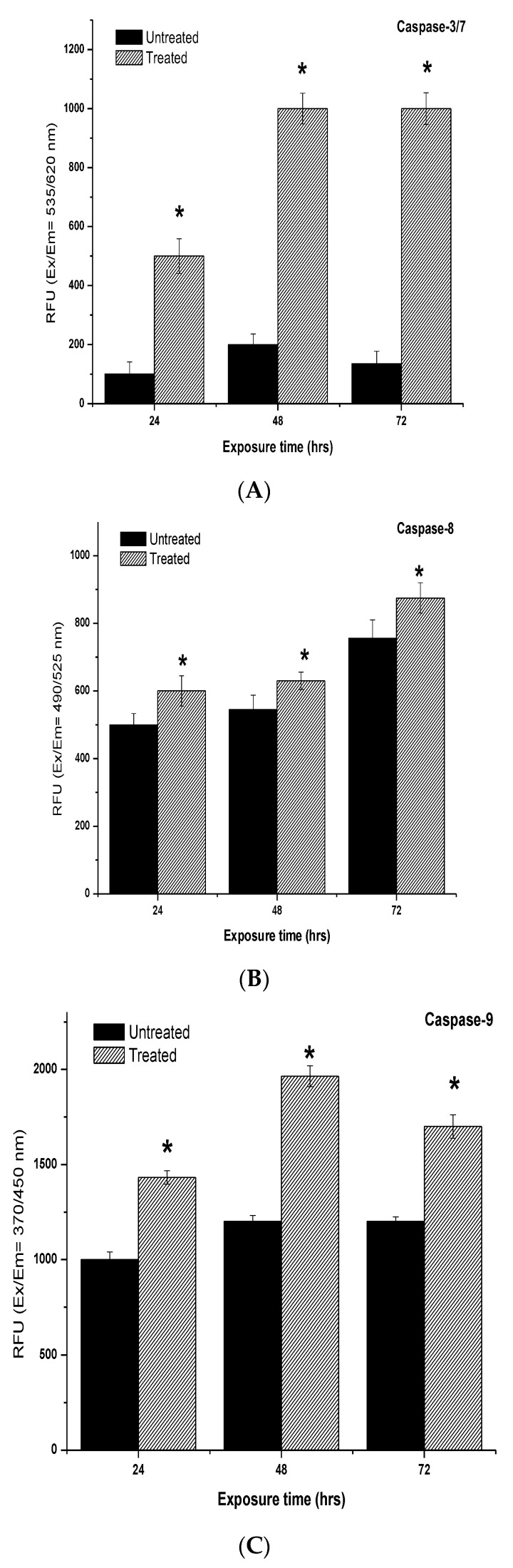
The caspase-3/7, -8 and -9 activities in MCF-7 cells treated for 24, 48 and 72 h. Each data point represents the mean of three independent experiments ± SD. * Significantly different from the control (*p* < 0.05). (**A**) 24 h, (**B**) 48 h and (**C**) 72 h of incubation.

Both intrinsic and extrinsic apoptotic pathways are characterized by the activation of caspases after treatment with the anti-cancer compounds. Therefore, in this study, we have determined the activities of caspases-3/7, -8 and -9. In the MCF-7 cell lines, 24 and 48 h of exposure to 7-geranyloxycinnamic acid resulted in a highly significant increase (by 500%) in caspase-3/7 enzyme activity (*p* < 0.01) when compared to the untreated control (Figure 24). Similarly, treatment with 7-geranyloxycinnamic acid for 24, 48, and 72 h caused a significant increase in caspase-8 enzyme activity compare to control. After 24 and 72 h of incubation with 7-geranyloxycinnamic acid, there are significant increases (*p* < 0.05) in caspase-9 enzyme activity. In comparison with the untreated MCF-7 cells, the caspase-9 enzyme activity increased after 48 h of treatment with 7-geranyloxycinnamic acid.

**Figure 25:**
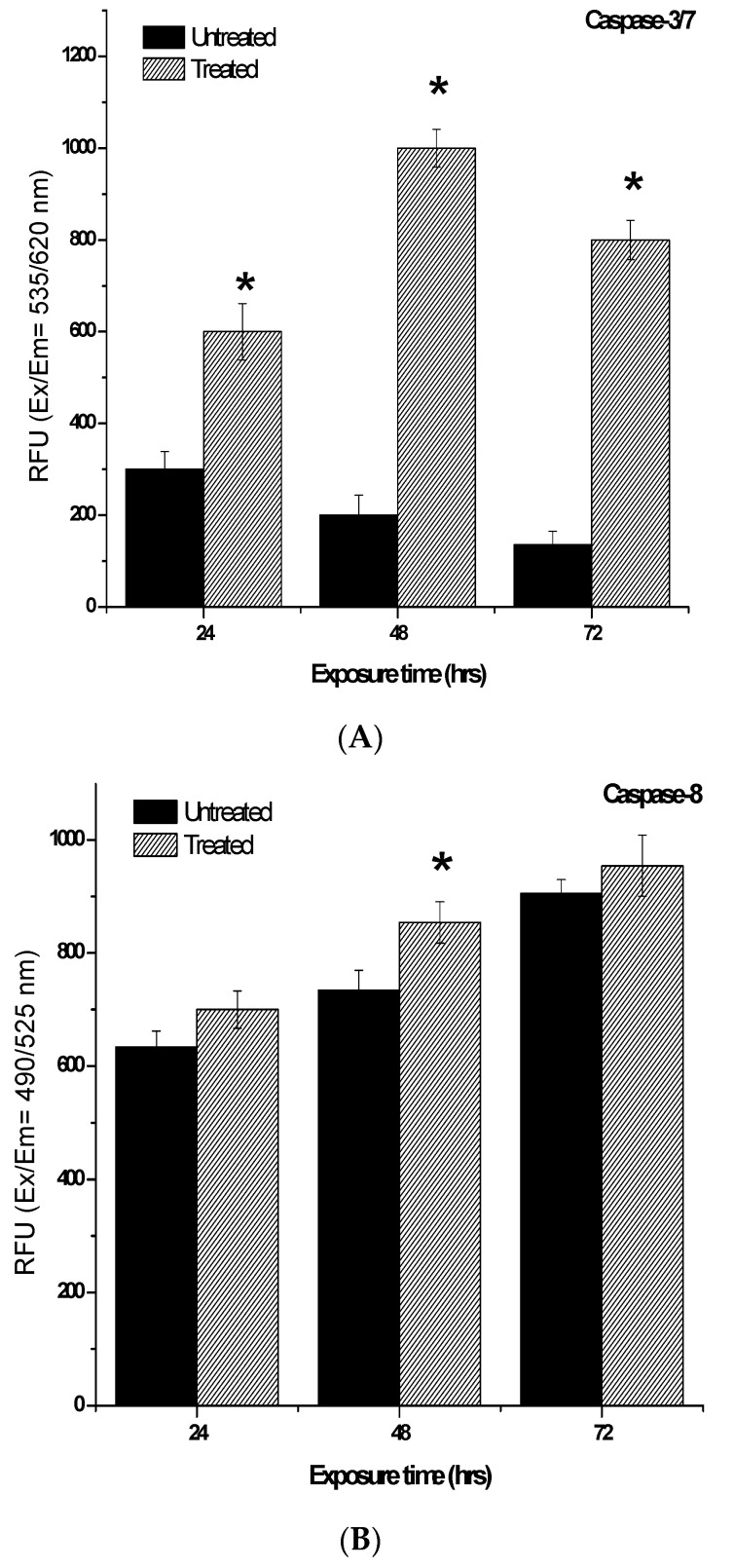

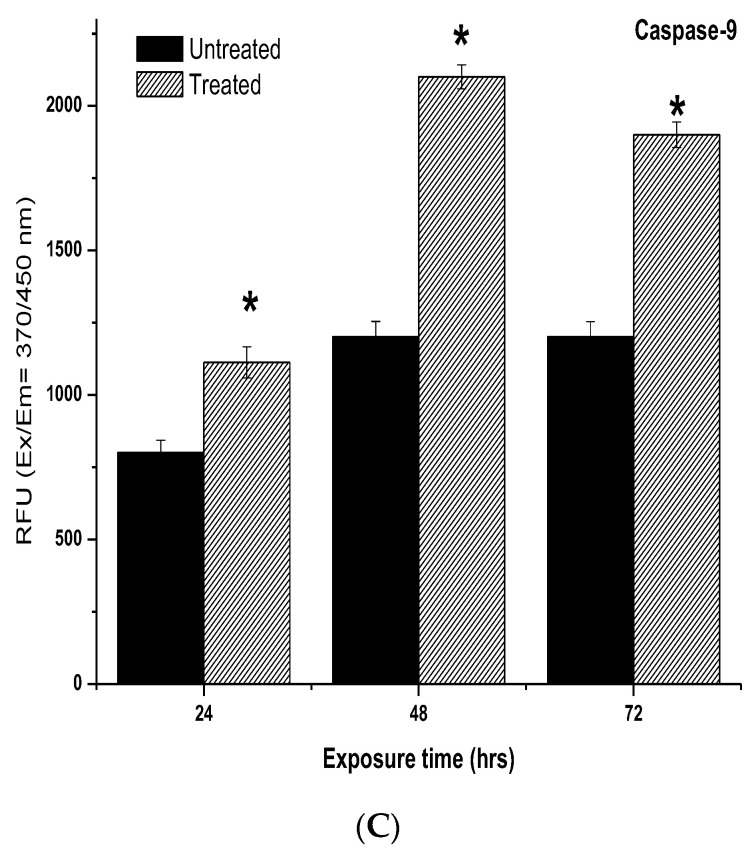
The caspase-3/7, -8 and -9 activities in MDA-MB-231 cells treated for 24, 48 and 72 h. Each data point represents the mean of three independent experiments ± SD. * Significantly different from the control (*p* < 0.05). (**A**) 24 h, (**B**) 48 h and (**C**) 72 h of incubation.

In the case of the MDA-MB-231 cell line, treatment with IC_50_ concentrations of 7-geranyloxycinnamic acid significantly elevated the activity of caspase-3/7 by 2.0-, 5.0- and 5.9-fold after 24, 48, and 72 h of incubation (Figure 25). When compared to the untreated MDA-MB-231 cells, the caspase-8 enzyme activity increased but not significantly (*p* > 0.05) after 24 and 72 h of incubation with 7-geranyloxycinnamic acid to 10.4% and 5.4%, respectively (Figure 25). The highest caspase-8 enzyme activity (16.3%) of untreated cells was measured in the MDA-MB-231 cells treated with 7-geranyloxycinnamic acid for 48 h of incubation. After 24 and 48 h of incubation, the caspase-9 enzymatic activity in MDA-MB-231 cells treated with 7-geranyloxycinnamic acid increased to 139.1% and 175.0%, respectively. A further increase in exposure to 7-geranyloxycinnamic acid for up to 72 h did not cause any significant increase (*p* > 0.05) in caspase-9 enzymatic activity compared to 48 h of exposure.

## 3. Discussion

Plants are the richest source of structurally diverse cytotoxic phytochemicals. It is critical to evaluate the in vitro cytotoxic potential of phytochemicals for their successive use in clinical settings to combat multidrug resistance associated with cancer cells. Growing scientific data suggest that a large number of traditional medicinal plants have been reported to exhibit cytotoxic potential against a variety of cancer cell lines [23]. Therefore, this study investigated the cytotoxic activities of the leaves of *M. lunu-ankenda*, a traditional medicinal plant in Malaysia. Firstly, we prepared three different crude extracts of leaves using different solvents (petroleum ether, chloroform, and methanol) and screened for their cytotoxic efficacy against three different cancer cell lines (HepG2, HT-29, and MCF-7). The MTT assay has been extensively used in cancer research to determine the viability and proliferation of cells after exposure to toxic substances, as well as for the characterization of the toxicity of new drugs [24,25,26]. Moreover, the determination of half maximal inhibitory concentration (IC_50_) using MTT assay is critical for evaluating the pharmacological activities of chemotherapeutic agents against cancer [27]. From our MTT assay results, we calculated the IC_50_ concentrations for the three different crude extracts against three tested cancer cell lines (Table 3). Our results showed that cancer cells were most sensitive to treatment with petroleum ether extract. Moreover, the IC_50_ concentration of petroleum ether extract is low for all the tested cancer cell lines. Many researchers have demonstrated the relationship between the type of organic solvent and the biological activity exhibited by natural products. Previous studies also demonstrated the cytotoxic potential of petroleum ether extract compared to other solvent extracts [28].

The petroleum ether-based extracts rich in lipophilic compounds of high purity have been reported to demonstrate selective anti-cancer effects [29,30,31,32,33]. Therefore, we have further subjected this petroleum ether extract to fractionation using column chromatography and isolated a white needle-shaped crystalline compound. From the NMR and LC-MS data (Table 2 and Figure 2, Figure 3 and Figure 4), the isolated pure compound from the petroleum ether was identified as 7-geranyloxycinnamic acid. Previous studies have also isolated 7-geranyloxycinnamic acid as a major compound from the petroleum ether extract of leaves [16]. In our previous study, we have demonstrated the significant neuroprotective effects of 7-geranyloxycinnamic acid [13]. Therefore, in this study, we evaluated the cytotoxic effects of 7-geranyloxycinnamic acid against breast cancer cell lines (MCF-7 and MDA-MB231) and the HT-29 cell line.

Research findings from the MTT assay (Figure 8) clearly showed that all tested concentrations of 7-geranyloxycinnamic acid were found to be highly cytotoxic towards both breast cancer cell lines, but not the HT-29 cell lines. Other studies isolated 7-geranyloxycinnamic acid from the methanol extract, which demonstrated the most potent free radical scavenging ability but showed weak antiproliferative activity against human leukemic cell line HL60, in contrast to what we reported in our current study [34]. The 5-fluorouracil acid is a cytotoxic drug that is already in clinical use for the treatment of a variety of cancers [35,36,37,38,39,40]. 5-FU was scientifically proven to be cytotoxic to Hep G2 but not normal cell lines [41]. However, 5-fluorouracil is associated with adverse side effects causing gastrointestinal damage, especially in the mucosa, leading to changes in intestinal permeability, bacterial translocation, and immunity [42,43]. Therefore, turning toward drugs or compounds with fewer side effects, such as our purified 7-geranyloxycinnamic acid, is of current interest. Our research findings (Table 4 and Table 5) showed that the IC_50_ concentrations of 7-geranyloxycinnamic acid were lower when compared to those of the standard drug 5-fluorouracil against both breast cancer cell lines (MCF-7 and MDA-MB231). Except at higher concentrations (10 and 20 μg/mL), 7-geranyloxycinnamic acid did not exert significant cytotoxicity towards the normal cell line (MCF-10a). This further confirms the higher selective cytotoxic efficacy of 7-geranyloxycinnamic acid compared to that of 5-fluorouracil towards breast cancer cell lines. As a result of this, in the current study, we have further investigated the cytotoxic mechanism of action of 7-geranyloxycinnamic acid against the breast cancer MCF-7 and MDA-MB231 cell lines.

Apoptosis is the cells’ natural mechanism of highly regulated programmed cell death which helps in the elimination of unwanted cells. However, cancer cells escape apoptosis, resulting in the uncontrollable proliferation of cells. Hence, it is highly desirable to develop pro-apoptotic compounds that can direct apoptosis of cancerous cells without causing damage to the healthy or normal cells [44]. The important hallmarks of apoptosis include nuclear fragmentation and changes in the morphology of the cells. In view of this, first, we examined the morphology of breast cancer cell lines using a phase contrast microscope. However, some morphological changes that enable the elucidation of the cell death process and the differentiation between necrosis and apoptosis may not be seen through optical microscopy, requiring other techniques such as electron microscopy [45,46]. To further analyze the morphology of the cell lines tested in this work, we additionally used a fluorescence microscope, scanning electron microscopy, and transmission electron microscope. Our SEM and TEM studies revealed the presence of structures resembling vacuoles in the cytoplasm of the cells (Figure 11) and cytoplasmic fragmentation (Figure 12). These changes became more evident with increasing exposure duration and resembled those induced in the human melanoma cell line (HT-144) after two days of incubation with cinnamic acid, a pro-apoptotic substance closer to our compound [18].

The dual labeling of acridine orange and propidium iodide, as well as Annexin V/PI staining, aids in the observation of apoptotic processes. Propidium iodide and acridine orange designate anatomically normal cells and apoptotic cells, respectively, by fluorescing from green to yellow and red when bound to DNA and cell membrane, respectively [47]. Annexin-FITC/PI double staining allows the discrimination between viable cells (FITC−/PI−), cells that are in an early stage of apoptosis (FITC+/PI−), late apoptotic cells (FITC+/PI+) and cells in necrosis (FITC−/PI+) [48,49,50,51,52,53]. Similar to what was observed in the present investigation, cinnamic acid and its derivative have been found to cause apoptotic characteristics [54,55]. When compared to the negative control, the results of flow cytometry revealed a much higher number of lysed cells (containing fragmented nucleic acid), supporting the findings of cytometric assays using Annexin V and propidium iodide. In contrast to the untreated control, 24 h of exposure to 7-geranyloxycinnamic acid at IC_50_ concentrations did not appear to cause any appreciable morphological alterations in either of the breast cancer cell lines. However, a longer duration of exposure to 7-geranyloxycinnamic acid at IC_50_ concentrations resulted in various alterations, such as cell retraction and a reduction in the interaction between nearby cells and necrotic and different phases of apoptotic cells. These findings suggest a possible cytostatic property of our compound after 24 h of incubation, while the increase in exposure time exhibits cytotoxic activity on both breast cancer cell lines. Our qualitative microscopic findings (SEM, TEM and fluorescent microscopic analyses) are in good agreement with the quantitative flow cytometric data, as they all indicate that our test chemical may have lethal effects by triggering apoptotic characteristics in both breast cancer cell lines.

The cell cycle analysis by flow cytometry uses markers which specifically bind to genetic material, providing exact information on the amount of DNA present at each phase of the cell cycle [56,57]. Therefore, any damage to cellular DNA causes the cell cycle to stop at precise check points so that the DNA can be repaired; if the damage cannot be repaired, the cell enters apoptosis and dies [58]. As a results, cell cycle analysis using flow cytometry enables a detailed study of the percentage of cells in different phases of mitotic cell division. Treatment with 7-geranyloxycinnamic acid caused alterations in the cell cycle of both breast cancer cell lines. When MCF-7 cells were exposed to 7-geranyloxycinnamic acid for 24 and 72 h at IC_50_ concentrations, the cell cycle was arrested at the G2/M and G0/G1 phases. Additionally, after two days of incubation, a marginally significant increase in the apoptotic sub-G1 fraction was found in this line. After incubating MDA-MB-231 cells with 7-geranyloxycinnamic acid at IC_50_ concentrations for 24 and 72 h, the population of these cells accumulated in the S-phase and G0/G1 phase. Although their mechanism of action is not entirely understood, previous studies have shown that several compounds related to cinnamic acid can induce cell cycle arrest at the S-phase, trigger apoptosis, and exert genotoxic effects in the human breast cancer cell lines [59].

Accumulation of intracellular ROS causes growth inhibitory effect. We used a fluorescence experiment using H2DCF-DA to determine if the 7-geranyloxycinnamic acid’s mode of action was dependent on the intracellular ROS. In the presence of intracellular ROS, dichloro-fluorescein (DCF) (fluorescent) is produced when intracellular esterase cleaves H2DCFDA into a non-fluorescent form (DCFH) [19]. Several investigators have reported the cytotoxic effect of cinnamic acids against different cancer cells of epithelial origin by inducing the ROS accumulation [60,61]. Previous studies also indicate that ROS-mediated DNA damage can induce apoptosis of cancer cells using plant-derived chemicals [62,63,64]. As evident from Figure 22 and Figure 23, treatment with 7-geranyloxycinnamic acid resulted in a significant increase in the accumulation of ROS in both breast cancer cell lines compared to the control. This suggests that the underlying mechanism of anti-proliferative activity of 7-geranyloxycinnamic acid is related to DNA cleavage or DNA intercalation mechanisms via interaction with ROS. It was also suggested that ROS can activate some proteins involved in the apoptotic process and oxidative stress, thereby causing cell death [65,66,67]. Previous studies also demonstrated that treatment with hydroxycinnamic acid could induce intrinsic pathway of apoptosis through the disruption of the mitochondrial membrane potential and induction of intracellular reactive oxygen species, thereby causing the death of cancer cells [68,69].

Undoubtedly, there is a need for the development of chemotherapeutic agents that specifically activate apoptotic signaling pathways (either extrinsic and intrinsic or mitochondrial apoptotic pathways) in cancer cells, thus providing opportunity for the development of targeted therapies for cancer treatment. Apoptosis is mediated by caspases, including initiator caspases (caspase-2, -8, -9, 10) and executioner caspases (caspase-3, -6, -7), which are a class of proteases that cleave a variety of cytoplasmic or nuclear proteins, leading to the apoptotic morphological changes and, ultimately, the death of the cells [24]. A previous study reported that cinnamic acid derivatives can induce apoptosis through the intrinsic pathway in the MDA-MB-231 and MCF-7 cells [59], which is in agreement with our study, wherein we showed that 7-geranyloxycinnamic acid can induce apoptotic cell death of the breast cancer MDA-MB-231 and MCF-7 cell lines. Another study also reported that the extract of *Cinnamomum cassia,* which is rich of cinnamic acid derivatives, showed significant cytotoxicity against human breast cancer cell lines via intrinsic and extrinsic pathways by increasing the activity of caspase-9 and -8, suggesting mitochondrial and death ligands’ involvement in apoptotic signaling [70]. In the present study, we showed that treatment with 7-geranyloxycinnamic acid significantly increased the activities of caspase-3/7/8 and 9, suggesting the possible activation of extrinsic and intrinsic apoptotic pathways, respectively. This points to the fact that 7-geranyloxycinnamic acid exerts significant cytotoxic effects by inducing apoptotic cell death in both the MDA-MB-231 and MCF-7 cell lines.

## 4. Materials and Methods

### 4.1. Plant Collection, Extraction, and Fractionation

The *Melicope lunu-ankenda* (Gaertn.) T. G. Hartly leaves were collected from the Biodiversity Unit, Institute of Bioscience (IBS) at Universiti Putra Malaysia (UPM). The plant was identified and authenticated by Dr. Shamsul Khamis and the voucher specimen (number SK2873/15) was deposited at the Herbarium IBS, UPM. Melicope lunu-fresh ankenda’s leaves were shade-dried at room temperature for about two weeks before being milled into a fine powder. The leaf powder material was then extracted three times from each solvent using increasing polarity solvents in the following order: petroleum ether, chloroform, and methanol for a total of 72 h. The filtrate from each extract was then concentrated using a rotary evaporator at 40 °C with reduced pressure using Whatman filter paper (0.45 m).

Using C18 reverse phase silica gel (40–63 m) (LiChroprep^®^, Germany), column chromatography was carried out. Three grams of crude petroleum ether extract and three grams of silica gel were placed within the chromatography column. The extract was then separated using a mobile phase combination of (50:50) methanol and water. Thin layer chromatography (TLC) was used to evaluate the fractions. The similar fractions were combined and further purified on silica gel 60 (0.04–0.06 mm) (Merck, Germany), which eluted with hexane gradually increasing in ethyl acetate as a mobile phase system. The compound collected were white needle-shaped crystals (112 mg). The following formula was used to calculate the percentage of yield.

% Yield = Weight of pure compound ÷ weight of the crude extract × 100(1)

### 4.2. Structure Characterization and Identification of 7-Geranyloxycinnamic Acid

#### 4.2.1. Nuclear Magnetic Resonance Spectroscopy

^1^H NMR and ^13^C NMR spectra were obtained on a 500 MHz Varian NMR spectrometer (USA) and reported in deuterated chloroform (CDCl_3_). The system was operated using tetramethyl silane (TMS) as an internal standard and the chemical shifts were expressed in parts per million (ppm), while coupling constant J signals were measured in hertz.

#### 4.2.2. Liquid Chromatography–Mass Spectrometry

The molecular mass of the compound was recorded using mass spectrometry (Agilent 6550 iFunnel Q-TOF LC-MS, Santa Clara, CA, USA) in high resolution of electron spray mode ESI in a positive mode. The sample was injected at a flow rate 0.1 mL/min and eluted with 0.1% formic acid in methanol as a mobile phase.

### 4.3. Cell Culture

All cell lines, including the non-tumorigenic human breast cell line (MCF 10 A), triple negative breast cancer (MDA MB 231), estrogen-positive breast cancer (MCF-7), human hepatocellular carcinoma (HepG2), and human colon adenocarcinoma cell line (HT29), were obtained from the MAKNA Cancer Research Laboratory, IBS, UPM. The Dulbecco minimum essential media (DMEM) (Nacalai Tesque Inc., Kyoto, Japan) supplemented with 10% fetal bovine serum (FBS), 1% 100 IU penicillin, and 100 g/mL streptomycin was used to maintain all cell lines (Nacalai Tesque, Japan). The cells were grown at 37 °C with 5% CO_2_ in a humidified environment. They performed routine trypsinization (0.05% Trypsin-EDTA) at 70–80% confluence; the cultures were continually maintained.

#### 4.3.1. Cell Viability Assay

The cell viability assay was conducted using 3-(4,5-dimethylthiazol-2-yl)-2,5-diphenyltetrazolium bromide (MTT) as described by Mosmann (1983) with some modifications [24]. Briefly, cells were seeded in 96-well flat-bottom tissue culture plate at a density of 1.0 × 10^5^ cell/well. Plates were incubated at 37 °C for 24 h to allow cells to attach to the bottom. Then, cells were treated with various concentrations of each of the crude extracts (200, 100, 50, 25, 12.5, 6.25 and 3.125 µg/mL) or 7-geranyloxycinnamic acid (20, 10, 5, 2.5, 1.25, 0.625, and 0.3125 µg/mL) or 5 fluorouracil (100, 50, 25, 12.5, 6.25, 3.125, and 1.5625) incubated at the time points indicated. At the end of the treatment period, 20 µL of MTT solution (5 mg/mL) was added to each well and the plate was incubated in the dark at 37 °C in a humidified atmosphere of 5% CO_2_ for approximately 4 h. Then, the MTT solution was aspired off and 100 µL of dimethyl sulfoxide (DMSO) was added to each well to solubilize the formazan crystals formed in each well. An ELISA plate reader (BioTek Synergy, Winooski, VT, USA) was used to read the absorbance at 570 nm. All the experiments were conducted in triplicate for each cell type. The selectivity index (SI) was then calculated to assess the cytotoxic selectivity of the tested compound and calculated as follows:

$$SI=\frac{IC50ofNormalcellline}{IC50ofCancercells}$$

where SI > 2 is indicated as highly selective as suggested by Machana et al. [71].

#### 4.3.2. Qualitative Assessment of Cell Morphology Using Phase Contrast Microscopy

Briefly, cells were seeded into a 6-well plate at a density of 1 × 10^5^ cell/well and incubated for 24 h at 37 °C in a humidified atmosphere of 5% CO_2_. After incubation, cells were treated with the pure compound (7-geranyloxycinnamic acid) and incubated for three different time points (24, 48 and 72 h). Following incubation, the qualitative assessment of cell morphology was carried out using phase contrast microscopy.

#### 4.3.3. Acridine Orange and Propidium Iodide (AO/PI) Staining

The cells were seeded into a 6-well plate at a density of 1 × 10^5^ cells/well and incubated for 24 h at 37 °C in a humidified atmosphere at 5% CO_2_. Then, cells were treated with IC_50_ of the compound for 24, 48 and 72 h. After incubation, cells were washed with phosphate buffer saline (PBS) twice and harvested via trypsinization. Cells were then resuspended in 200 μL of PBS and mixed gently. Then, an aliquot of cells (10 μL) was incubated with 10 μL of a mixture of equal volumes of AO and PI in dark. The mixture was then loaded onto a glass slide and covered with cover slip and observed within 30 min under the fluorescent microscope (Zeiss Axio, Dublin, CA, USA).

#### 4.3.4. Transmission Electron Microscopy (TEM)

The cells were treated with 7-geranyloxycinnamic acid as described above. After the treatment periods, cells were washed twice with PBS and harvested through centrifugation at 1000× *g* for 10 min at 4 °C. Then, cells were fixed with 4% glutaraldehyde and 1% osmium tetroxide for 6 and 2 h, respectively, at 4 °C. Following fixation, cells were washed three times for 10 min each with 0.1 M sodium cacodylate buffer; finally, the cell pellet was harvested by centrifuging at 3000× *g* for 5 min. The cells were further dehydrated with acetone concentrations of 35, 50, 75, and 95% for 10 min each. Three additional dehydration sessions using 100% acetone each lasted 15 min. A 1:1 acetone:resin solution was then applied to the cells for 60 min, a 1:3 solution for 120 min, and lastly, a 100% resin solution was applied overnight. The cells were then embedded by inserting these into a resin-filled beam capsule. The specimen was polymerized for two days in an oven at 60 °C and cut using an ultramicrotome into 1 µm thick sections which were stained with toluidine blue before cutting further into thinner sections of 60 to 90 nm. The thin sections were stained with uranyl acetate and lead for 15 and 10 min, respectively, before viewing under 11-7100 TEM (Hitachi, Tokyo, Japan) transmission electron microscope.

#### 4.3.5. Scanning Electron Microscopy (SEM)

The cells were treated as described above for 24, 48 and 72 h. Negative control cells were treated with 0.1% (*v/v*) DMSO for the same period of time. After the treatment period, the cells were collected by centrifuging at 1000× *g* for 10 min at 4 °C after being washed twice with PBS. Then, cells were fixed with 4% glutaraldehyde and 1% osmium tetroxide for 6 and 2 h, respectively, at 4 °C. After fixation, cells were washed three times with 0.1 M sodium cacodylate buffer (10 min each) by centrifuging at 3000× *g* for 5 min. The cells were then dehydrated successively for 10 min with 35, 50, 75 and 95% acetone. Cells were further dehydrated three times with 100% acetone for 15 min each, and then they were dried on a critical drier for around 30 min. The cell pellets were coated with gold particles, placed on stubs, and viewed with a JSM 6400 scanning electron microscope (Jeol, Peabody, MA, USA).

#### 4.3.6. Annexin V-FITC Apoptosis Detection Assay

According to the manufacturer’s protocol, cell death was detected using an Annexin V-FITC apoptosis detection kit (BD Pharmingen, Becton, NJ, USA). Cells were treated with the pure compound at the indicated treatment periods. Then, cells were harvested using trypsinization and PBS wash; finally, these were re-suspended in binding buffer (0.1 M HEPES/NaOH pH 7.4, 1.4 M NaCl and 25 mM CaCl_2_). A mixture of 5 µL Annexin V-FITC and PI each was added to 40 µL cell suspension and incubated at room temperature in the dark for 15 min. Then, 450 μL of binding buffer was added to the stained cells, vortexed, and analyzed using a flow cytometer (BD FACS, Calibur, San Francisco, CA, USA).

#### 4.3.7. Intracellular Reactive Oxygen Species (ROS) assay

ROS levels were determined using an OxiSelect intracellular ROS assay kit according to the manufacturer’s protocol. Following treatment with the pure compound, cells were harvested through trypsinization and washed with an ice-cold phosphate buffer solution (PBS) at 1000 rpm for 5 min at 4 °C. The resulting cell pellets were re-suspended in a cold 100 µL ROS staining solution (OxiSelect, Cells Biolabs) and incubated in a humidified atmosphere of 5% CO_2_ at 37 °C for one hour. After incubation, cells were analyzed using flow cytometry (BD FACS, Calibur, San Francisco, CA, USA).

#### 4.3.8. Cell Cycle Assay

Cell cycle Assay Kit Green Fluorometric (Abcam, UK) was used for the cell cycle analysis, and the MCF-7 and MDA-MB 231 cells were seeded in accordance with the protocol’s instructions. Nuclear Green CCS1 was added to 0.5 mL of cell suspension after treatment with the pure substance, and the mixture was then incubated at 37 °C for 30 to 60 min. The samples were analyzed with a flow cytometer (BD FACS, Calibur, San Francisco, CA, USA) using the FL1 channel.

#### 4.3.9. Measurement of Caspase-3/7, -8, and -9 Activity

Using the Multiplex Activity Assay Kit (Fluorometric), the activities of caspases 3, 8, and 9 were assessed (Abcam, UK). A 2 × 10^4^ cells/90 µL per well were seeded in to a sterile 96-well plate and incubated overnight. Following treatment with the pure compound, caspase assay solution was added and incubated at room temperature for 30–60 min, and fluorescence intensity was monitored at the given excitation and emission wavelengths for each caspase (Ex/Em = 535/620 nm, Caspase 3; Ex/Em = 490/525 nm, Caspase 8; Ex/Em = 370/450 nm, Caspase 9).

### 4.4. Statistical Analysis

All experiments were performed in triplicate and the results are expressed as mean ± SD. Data analysis was performed using GraphPad Prism 5.0 (GraphPad Software Inc., La Jolla, CA, USA). One-way analyses of variance were performed followed by Tukey’s post hoc tests to compare replicate means of treatment and control groups. The significance was set at *p* < 0.05.

## 5. Conclusions

There has been an increase in interest in recent years in discovering new anticancer drugs that can effectively and selectively kill cancer cells with fewer/no toxic effects against normal cells. In this context, compared to the currently available anticancer medications that are linked to unfavorable side effects, plant-derived drugs have emerged as the most alternative form of treatment for cancer patients. *Melicope lunu-ankenda* is an evergreen shrub in tropical countries and is known for its clinical and edible properties. The results of the present investigation demonstrated the low IC_50_ cytotoxicity of 7-geranyloxycinnamic acid extracted from *M. lunu-ankenda* leaves against various cancer cell lines. Our research results also indicate the ability of 7-geranyloxycinnamic acid to induce programmed cell death of breast cancer cell lines via both the extrinsic and intrinsic pathways. The enhanced production of caspases and cell cycle arrest may play a role in the cytotoxic action of 7-geranyloxycinnamic acid. In the cancer cell line MCF-7, 7-geranyloxycinnamic acid can cause G0/G1 and G2/M cell cycle arrest as well as a reduction in Sub G0/G1 phase. In contrast, 7-geranyloxycinnamic acid stops the cell cycle of MDA-MB-231 cells in the G0/G1 phase. Taken together, we conclude that 7-geranyloxycinnamic acid might have possible anti-cancer effects; however, future studies are still required to further evaluate its anti-cancer potential in vivo.

## Figures and Tables

**Table 1:** The weight and percentage yield of crude extracts from *M. lunu-ankenda leaves*.

Dried Material (g)	Weight and % Yield of Petroleum Ether Extract	Weight and %Yield of Chloroform Extract	Weight and %Yield of Methanol Extract
960 g	61.62 g and 6.48%	41.93 g and 4.37%	102.16 g and 10.64%

**Table 2:** ^1^H NMR and ^13^C NMR data for the isolated compound.

Carbon Position	Isolated Compound		Reference (Hashim et al., 2005)	
	δ_C_ (ppm) in CDCL_3_	δ_H_ (J in Hz)	δ_C_ (ppm) in DCL_3_	δ_H_ (J in Hz)
1	172.4	-----	172.68	-----
2	114.5	6.33 (1H, d, 15 Hz)	114.38	6.26 (1H, d, 16)
3	146.7	7.75 (1H, d, 15 Hz)	146.8	7.72 (1H, d, 16)
4	126.6	-----	126.57	-----
5,9	130.1	7.50 (2H, d, 10 Hz)	130.07	7.48 (2H, d, 8.25)
6,8	115.1	6.93 (2H, d, 10 Hz)	115.07	6.91 (2H, d, 8.25)
7	161.1	-----	161.08	-----
1′	65.0	4.58 (2H, d, 5 Hz)	64.99	4.58 (2H, d, 6.4)
2′	118.9	5.49 (1H, t, 5 Hz)	118.88	5.47 (1H, t, 6.4)
3′	141.8	-----	141.77	-----
4′	39.5	2.10 (2H, d, 5 Hz)	39.5	2.10 (2H, m)
5′	26.2	2.14 (2H, t, 10 Hz)	26.23	2.10 (2H, m)
6′	123.7	5.10 (1H, t, 10 Hz)	123.66	5.07 (1H, t, 7.8)
7′	131.9	-----	131.88	-----
8′	17.7	1.60 (3H, s)	16.69	1.60 (3H, s)
9′	16.7	1.74 (3H, s)	17.69	1.74 (3H, s)
10′	25.7	1.67 (3H, s)	25.68	1.67 (3H, s)

d: doublet peaks; m: multiple peaks; t: triplet peaks.

**Table 3:** Calculated IC_50_ values of petroleum ether, chloroform and methanol crude extracts for different cell lines.

Extract	HepG2	MCF-7	HT-29
Petroleum ether	75.181 ± 0.011	76.107 ± 0.011	20.645 ± 0.023
Chloroform	152.809 ± 0.150	76.199 ±0.038	19.662 ± 0.013
Methanol	77.012 ± 0.007	89.332 ± 0.031	181.020 ± 0.043

**Table 4:** IC_50_ values of 7-geranyloxycinnamic acid and 5-fluorouracil on human breast cancer MDA-MB231, human colon cancer HT29, human breast cancer MCF-7, and human normal breast MCF-10a cells after 24, 48, and 72 h of incubation.

Cell Lines	Cell Lines (IC_50_ in µg/mL)											
	MDA-MB231			HT29			MCF-7			MCF-10a		
Incubation (h)/Compound	24	48	72	24	48	72	24	48	72	24	48	72
7-geranyloxycinnamic acid	5.368 ± 0.777	3.975 ± 0.857	1.732 ± 0.060	9.057 ± 0.596	6.797 ± 0.306	6.748 ± 0.522	4.936 ± 0.345	3.335 ± 0.728	1.847 ± 0.212	ND	94.201 ± 0.429	48.814 ± 0.386
5-fluorouracil	34.464 ± 1.541	4.445 ± 0.613	2.505 ± 0.448	34.904 ± 1.316	10.336 ± 0.610	7.952 ± 0.905	30.252 ± 1.156	4.446 ± 0.595	2.163 ± 0.606	NA		

ND: not detected; NA: not available.

**Table 5:** Selectivity index (SI) of 7-geranyloxycinnamic acid and 5-fluorouracil on human breast cancer MDA-MB231, human colon cancer HT29, human breast cancer MCF-7, and human normal breast MCF-10a cells after 24, 48, and 72 h of incubation.

**Test Compounds**	**IC_50_ (μg/mL)**		**Selectivity Index (SI)**
	**MCF-10a Cells**	**MDA-MB231 Cells**	
7-geranyloxycinnamic acid (24 h)	ND	5.368 ± 0.777	0
7-geranyloxycinnamic acid (48 h)	94.201 ± 0.429	3.975 ± 0.857	24
7-geranyloxycinnamic acid (72 h)	48.814 ± 0.386	1.732 ± 0.060	28
**Test Compounds**	**IC_50_ (μg/mL)**		**Selectivity Index (SI)**
	**MCF-10a Cells**	**MCF-7 Cells**	
7-geranyloxycinnamic acid (24 h)	ND	4.936 ± 0.345	0
7-geranyloxycinnamic acid (48 h)	94.201 ± 0.429	3.335 ± 0.728	28
7-geranyloxycinnamic acid (72 h)	48.814 ± 0.386	1.847 ± 0.212	26

ND: not detected.

## Data Availability

Not applicable.

## Supplementary Materials

The following supporting information can be downloaded at: https://www.mdpi.com/article/10.3390/molecules28083612/s1, Figure S1: gCOSY NMR spectrogram; Figure S2: gHSQCAD NMR spectrogram QC; Figure S3: gHMBCAD spectrogram.

## Sample Availability

Sample of the compound is available from the authors.
