# Supplementary material for: 7-Geranyloxycinnamic Acid Isolated from Melicope lunu-ankenda Leaves Perturbs Colon Cancer and Breast Cancer Cell Lines’ Growth via Induction of Apoptotic Pathway
Source: Molecules. 2023 Apr 21;28(8):3612. doi: 10.3390/molecules28083612 (PMC10142869; doi:10.3390/molecules28083612)
Supplement: Supplementary file 1 [file molecules-28-03612-s001.zip › molecules-2032691-supplementary.pdf]

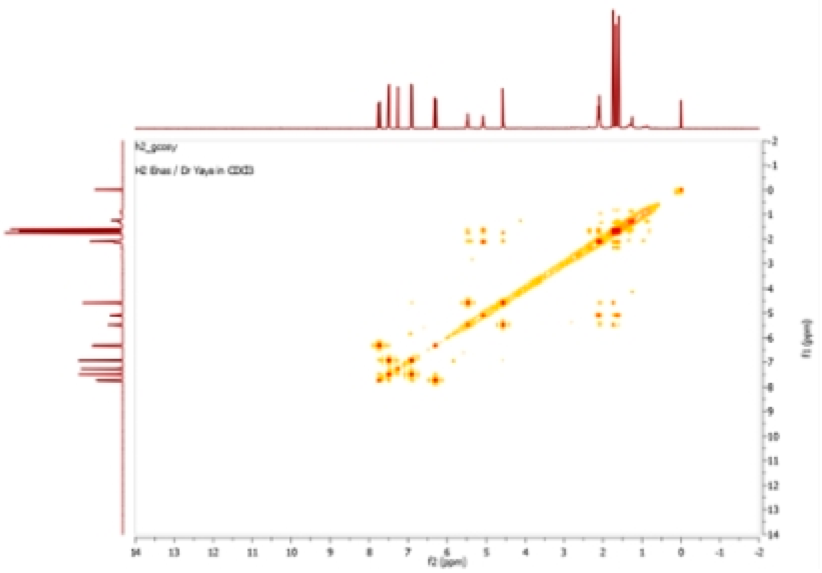

Figure S1: gCOSY NMR spectrogram

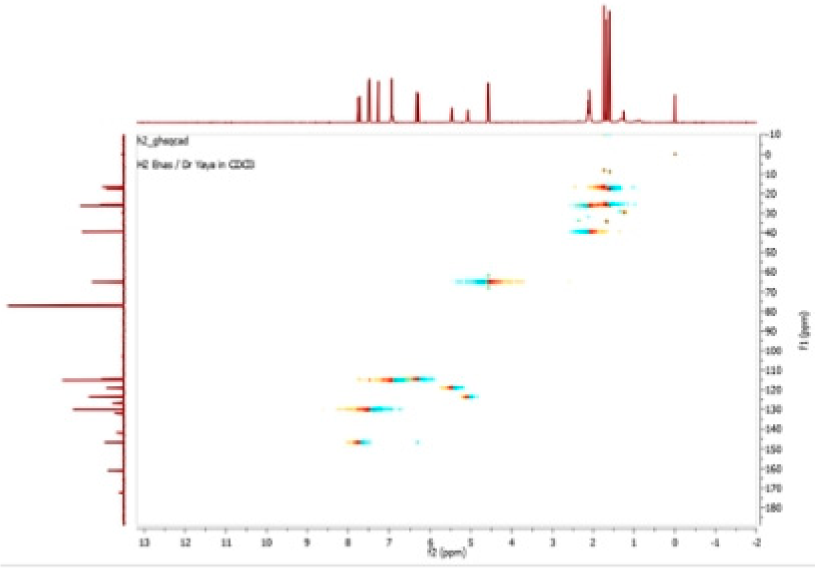

Figure S2: gHSQCAD NMR spectrogram QC

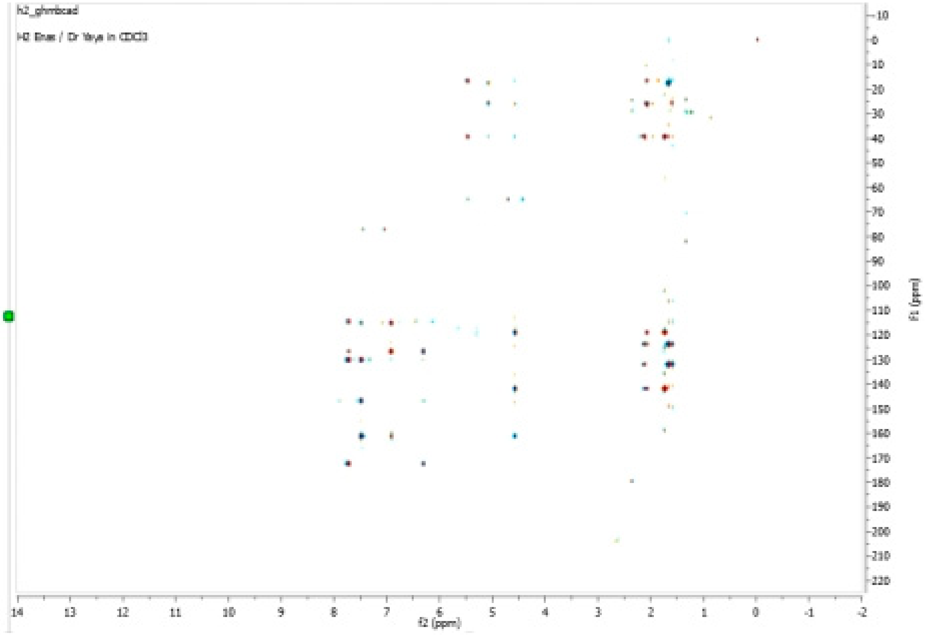

Figure S3: gHMBCAD spectrogram.
